# Rice grain nutritional traits and their enhancement using relevant genes and QTLs through advanced approaches

**DOI:** 10.1186/s40064-016-3744-6

**Published:** 2016-12-09

**Authors:** Anumalla Mahender, Annamalai Anandan, Sharat Kumar Pradhan, Elssa Pandit

**Affiliations:** Crop Improvement Division, ICAR-National Rice Research Institute (Formerly, Central Rice Research Institute), Cuttack, Odisha 753006 India

**Keywords:** Grain nutritional properties, Grain nutraceutical properties, Grain vitamins and minerals, Grain phytic acid, Grain protein, Grain amino acid, Grain phenolic and flavonoid compounds, Molecular markers

## Abstract

**Background:**

Rice breeding program needs to focus on development of nutrient dense rice for value addition and helping in reducing malnutrition. Mineral and vitamin deficiency related problems are common in the majority of the population and more specific to developing countries as their staple food is rice.

**Results:**

Genes and QTLs are recently known for the nutritional quality of rice. By comprehensive literature survey and public domain database, we provided a critical review on nutritional aspects like grain protein and amino acid content, vitamins and minerals, glycemic index value, phenolic and flavonoid compounds, phytic acid, zinc and iron content along with QTLs linked to these traits. In addition, achievements through transgenic and advanced genomic approaches have been discussed. The information available on genes and/or QTLs involved in enhancement of micronutrient element and amino acids are summarized with graphical representation.

**Conclusion:**

Compatible QTLs/genes may be combined together to design a desirable genotype with superior in multiple grain quality traits. The comprehensive review will be helpful to develop nutrient dense rice cultivars by integrating molecular markers and transgenic assisted breeding approaches with classical breeding.

## Background

Rice is the most well known cereal and staple food which serves as major carbohydrate for more than half of the world population. Half of the world’s population is suffering from one or more vitamin and/or mineral deficiency (World Food Program 2015). More than three billion people are affected by micronutrient malnutrition and 3.1 million children die each year out of malnutrition (Gearing 2015) and the numbers are gradually increasing (FAO 2009; Johnson et al. 2011). The developed countries are managing deficiency by adopting fortification programs, but same programs are not affordable to poor countries. Therefore, an alternative and less expensive strategy is to modify the nutritional quality of the major cereals consumed by the people. To improve the nutritional value of rice, research programs should be reoriented to develop high yielding cultivars with nutrient dense cultivars either by selective breeding or through genetic modification (Gearing 2015). Increase in literacy percentage and awareness of diet, people tend to be more health conscious and interested to have nutritionally enriched food. The quality of rice is an important character to determine the economic value in the export market and consumer acceptance (Pingali et al. 1997).

**Fig. 1:**
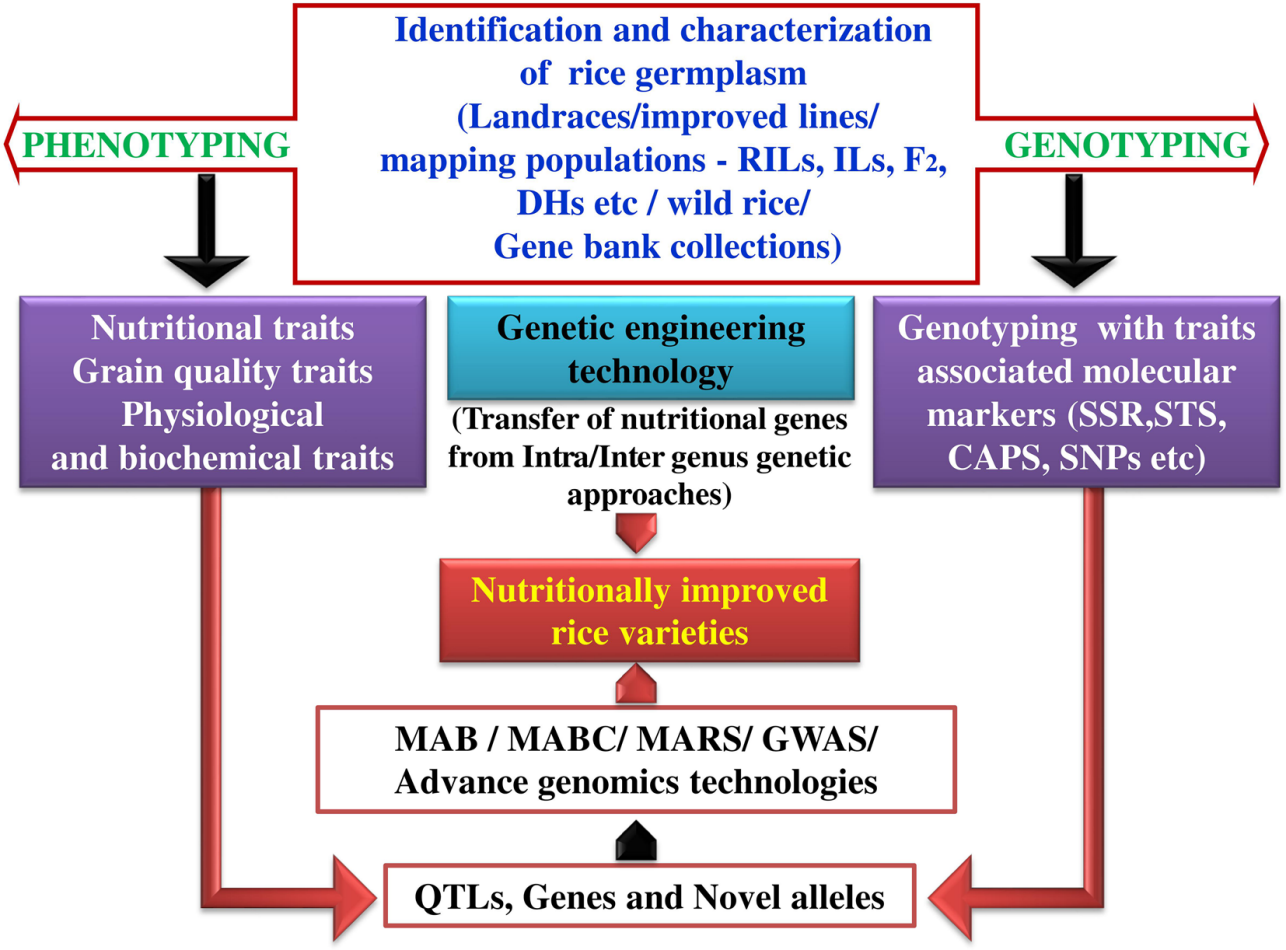
Integration of phenotypic and molecular breeding approaches for improvement of neutraceutical properties in rice grain

The genetic basis of the accumulation of micronutrients in the grain, mapping of the quantitative trait loci (QTL) and identification of genes will provide the basis for preparing the strategies and improving the grain micronutrient content in rice. Integrating marker assisted breeding with classical breeding makes, the possibility to track the introgression of nutritional quality associated QTLs and genes into a popular cultivar from various germplasm sources (Fig. 1). Till date classical breeding has a significant impact on improving biofortification of rice cultivars by making crosses, backcrosses and selection of the desired superior rice cultivars with high nutritional value. However, by availing technologies such as DNA markers, genetic engineering and allele mining offers an opportunity to use them as a tool to detect the allelic variation in genes underlying the traits and introgression of nutrition related QTLs/genes to improve the efficiency of classical plant breeding via marker-assisted selection (MAS).

Molecular markers such as SNPs (Ohstubo et al. 2002; Bao et al. 2006; Bert et al. 2008; Mammadov et al. 2012), SSRs (Anuradha et al. 2012; Nagesh et al. 2013; Gande et al. 2014), STS (Chandel et al. 2011; Gande et al. 2014), etc. have been developed. Integration of the markers into the breeding programs for effective selection of the plants at early stage of crop growth provides an opportunity to achieve the target earlier than the classical breeding program. Genomic approaches are particularly useful when working with complex traits having multigenic and influence of environment. In this new plant breeding era, genomics will be an essential aspect to develop more efficient nutritional rich rice cultivars (Perez-de-Castro et al. 2012), for reducing human health problems relating to mineral nutrition. Therefore, this is an effective approach for future rice breeding to reduce the malnutrition. By availing the different molecular approaches and advanced genomic technologies such as SNPs array, genome sequencing, genome-wide association mapping, transcriptome profiling, etc. could be strategically exploited to understand molecular mechanism and their relation between the genotypes and phenotypic traits leading to development of improved rice varieties (Chandel et al. 2011; Varshney et al. 2014; Malik et al. 2016; McCouch et al. 2016; Peng et al. 2016).

## Traits for improvement of the grain nutritive value

In the present situation, attention on grain quality and nutritional value has become a primary thought for producers and consumers. Rice grain is relatively low in some essential micronutrients such as iron (Fe), zinc (Zn) and calcium (Ca) as compared to other staple crops like wheat, maize, legumes and tubers (Adeyeye et al. 2000). However, rice grain consists of ~80% starch and its quality is dependent on combination of several traits. Another component of nutritive value of rice is bran, an important source of protein, vitamins, minerals, antioxidants, and phytosterols (Iqbal et al. 2005; Liu 2005; Schramm et al. 2007; Renuka and Arumughan 2007). Rice bran protein has a great potential in the food industry, having unique nutraceutical properties (Saunders 1990) and reported as hypoallergenic food ingredient in infant formulations (Helm and Burks 1996) and having anti-cancer properties (Shoji et al. 2001). Improvement in these components in the grain can be useful to reduce malnutrition.

## Nutritional and nutraceutical properties of rice

### Grain protein and amino acid content

**Fig. 2:**
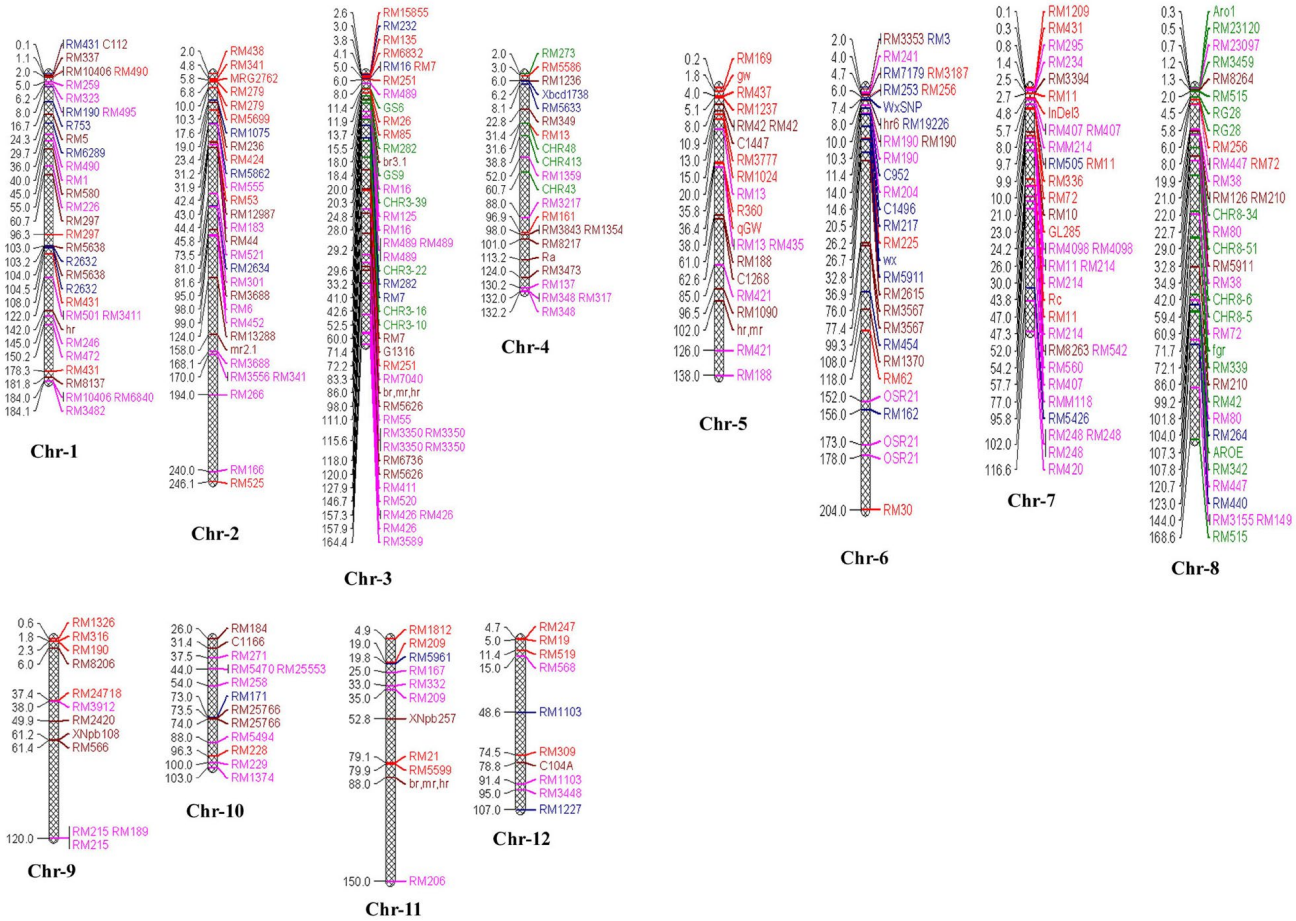
Depicted diagram of molecular marker positions associated with grain nutritional quality of rice distributed on 12 chromosomes from comprehensive literature survey. Molecular marker on right and their position (cM) on *left side* of the chromososmes. *MPGQ* milling properties of grain quality, *GA* grain appearance (*red*), *CP* cooking properties (*blue*), *NF* nutrition factors (*pink*), *FRG* fragrance of rice grain (*green*) (*colors* indicate markers related to nutritional quality traits in rice)

Protein energy malnutrition affects 25% of children where their dietary intake is mainly on rice and staple crops have low levels of essential amino acids (Gearing 2015). Therefore, attempts to improve the nutritional value of rice have been concentrated on protein content (PC) and other nutritional quality (Fig. 2). The amount of PC in rice is relatively low (8.5%) as compared to other cereals like wheat (12.3%), barley (12.8%) and Millet (13.4%) and an average of PC in milled rice is about 7 and 8% in brown rice. The total seed protein content of rice is composed of 60–80% glutelin and 20–30% prolamin, controlled by 15 and 34 genes respectively (Kawakatsu et al. 2008; Xu and Messing 2009). Rice supplies about 40% of the protein to human through diet in developing countries and quality of PC in rice is high, due to rich in lysine (3.8%) (Shobha Rani et al. 2006). Therefore, improvement of PC in rice grain is a major target for the plant breeders and biotechnologists. So far, by classical breeding effort, very limited success has been achieved because of the complex inheritance nature and the large effect of environment on protein content (Coffman and Juliano 1987). According to Iqbal et al. (2006), more than 170 million children and nourishing mothers suffered from Protein-calorie malnutrition (PCM) in developing Afro-Asian countries. In comparison with meat, plant proteins are much less expensive and nutritionally imbalanced because of their deficiency in certain essential amino acids (EAAs).

In general, cereal proteins are low in lysine (Lys 1.5–4.5 vs. 5.5% of WHO recommendation), tryptophan (Trp, 0.8–2.0 vs. 1.0%), and threonine (Thr, 2.7–3.9 vs. 4.0%). Pulses and most vegetable protein contain 1.0–2.0% of sulfur containing amino acid (methionine and cysteine), compared with the 3.5% of the WHO reference protein (Sun 1999). Therefore, these EAAs become the limiting amino acids in cereals and legumes. Recently, Han et al. (2015) compared the quality of rice bran protein (RBP) with animal and vegetable proteins. The digestibility of RBP (94.8%) was significantly higher than that of rice endosperm protein (90.8%), soy protein (91.7%) and whey protein (92.8%) which is same as that of casein. Among the total grain PC, rice bran protein appears to be a promising protein source with good biological value and digestibility.

Recently, Mohanty et al. (2011) reported 16.41 and 15.27% of crude protein in brown rice of ARC 10063 and ARC 10075 respectively on dry weight basis. They observed the total free amino acid content to be higher in these accessions and lysine content was positively correlated with the grain protein content in contrary to the view of Juliano et al. (1964) and Cagampang et al. (1966). Subsequently, by exploiting ARC 10075 as a donor, CR Dhan 310 (IET 24780) rice variety was developed with high protein content of 11% and rich in threonine and lysine (NRRI Annual Report 2014–2015). Several reports claim the varying levels of PC from 4.91 to 12.08%, lysine of 1.73–7.13 g/16 g N and tryptophan from 0.25 to 0.86 g/16 g N in rice accessions (Banerjee et al. 2010). Utilizing the efficiency of molecular marker technology, PC in brown and milled rice were mapped using various rice populations (Tan et al. 2001; Aluko et al. 2004; Weng et al. 2008; Zhang et al. 2008; Yu et al. 2009; Zhong et al. 2011; Yun et al. 2014).

### Vitamins and minerals

Forty-nine nutrients are required for normal growth and development and the demand is fulfilled by nutrients supplied by cereals, particularly rice (Welch and Graham 2004). Among these nutrients, mineral elements play beneficial role directly or indirectly in human metabolism. The wide spread occurrence of anemia and osteoporosis due to deficiency of iron and calcium respectively was observed in most developing countries as well as developed countries (Welch and Graham 1999). In the scenario, plant breeders started to pay more attention to improve the nutrient qualities especially mineral elements of major food grain crops (Zhang et al. 2004). Several researchers have reported genetic differences of mineral elements in rice (Gregorio et al. 2000; Zhang et al. 2004; Anandan et al. 2011; Ravindra Babu 2013; Jagadeesh et al. 2013). However, limited number of reports was observed for molecular level study and QTLs for vitamin and mineral content in rice. Brown rice is an important source of vitamins and minerals and by polishing the brown rice, several nutritional components such as dietary fiber, vitamins and phenols are eliminated that are beneficial to human health.

### Glycemic index value

Glycemic index (GI) is an indicator for the response of blood sugar levels based on the amount of carbohydrate consumption (after ingestion), which can be measured by rapidly available glucose (RAG). Rice, as a staple food contains 80% of starch and increased consumption leads to risk of type II diabetes (Courage 2010) and is predicted to affect almost 330 million people by 2030 (Misra et al. 2010). Brand-Miller et al. (2000) categorized glycemic index foods into low (GI value <55), medium (GI value 56–69) or high (GI value >70) GI foods. Recent studies have shown the ability of lower GI value will help to improve glycemic control in diabetics and cardiovascular diseases (Brand-Miller et al. 2003; Srinivasa et al. 2013). Low GI foods more slowly convert the food into energy by the body, thereby blood glucose levels become more stable than diets based on high GI foods. Therefore, identification of lower GI crops would play a major role in managing the disease. Thus, the diabetic sufferers in low-income countries such as Bangladesh, India, Indonesia, Malaysia and Sri Lanka may offer an inexpensive way for managing the disease (Fitzgerald et al. 2011). GI range may vary among the genotypes as well as the growing regions. GI varied from 54 to 121 among rice genotypes (Manay and Shadaksharaswamy 2001).

The degree of gelatinization is proportional to the amount of amylose; the less amylose there is, the greater the degree of gelatinization and vice versa. In other words, starches with lower amylose content will have higher Glycemic Indexes. Inversely, starches with a higher amylose content will be less susceptible to gelatinization, that is, to breaking down into glucose, that which makes for low Glycemic Indexes. The amount of amylose content (AC), *Waxy* haplotype and digestibility of rice are significantly correlated (Fitzgerald et al. 2011) and observed that AC plays a key role in rate of starch digestion and GI (Kharabian-Masouleh et al. 2012). Apparent amylose content is primarily controlled by the Waxy gene which codes for granule bound starch synthase (Chen et al. 2008a). The combination of two single-nucleotide-polymorphism (SNP) markers in the Waxy gene allows for the identification of three marker haplotypes in this gene. The first SNP is at the leader intron splice site (In1 SNP), and the second polymorphism is in exon 6. The haplotypes explained 86.7% of the variation in apparent amylose content and discriminated the three market classes of low, intermediate and high AC rice from each other.

Chen et al. (2008a, b), Larkin and Park (2003) and Kharabian-Masouleh et al. (2012) reported that *Waxy* gene showed four haplotypes viz., *In1T*-*Ex6A*, *In1G*-*Ex6C*, *In1G*-*Ex6A* and *In1T*-*Ex6C* used for the classification of AC in rice. Conversely, Cheng et al. (2012) identified intron1 is insufficient to explain the genetic variations of AC in rice. Therefore, the study based on the AC and molecular analysis would be helpful for the selection of appropriate nutritional quality rice for diabetic. Angwara et al. (2014) characterized 26 Thai rice varieties for RAG and Waxy haplotype (*In1*-*Ex6*) as GI indicators. The four haplotypes, classified 26 Thai rice varieties into grups consisting four varieties having G-A, nine varieties harboring G-C, 13 varieties carrying T-A or T-C allele associated with high, intermediate and low amylose respectively and the varieties having G-A haplotype exhibited low RAG.

### Phenolic and flavonoid compounds of rice grain

The phytochemicals such as phenolic compounds (tocopherols, tocotrienols and γ-oryzanol) and flavonoids (anthocyanidin) are responsible for good source of natural antioxidant and grain colour respectively. Kernel of red rice is characterized by the presence of proanthocyanidins whereas black rice is characterized by the accumulation of anthocyanins, mainly cyanidin-3-glucoside and peonidin 3-glucoside. These compounds help in decreasing the toxic compounds and reduce the risk of developing chronic diseases including cardiovascular disease, type-2 diabetes, reduction of oxidative stress and prevention of some cancers (Ling et al. 2001; Kong et al. 2003; Hu et al. 2003; Iqbal et al. 2005; Yawadio et al. 2007; Shao et al. 2011).

Red rice has phenolic compounds in the range of 165.8–731.8 mg gallic acid equivalent (GAE) 100 g^−1^ (Shen et al. 2009) and black/purple rice reported to have higher amount of Fe, Zn, Ca, Cu and Mg than red rice (Meng et al. 2005). On the other hand, pigmented rice reported to have higher amount of antioxidative activity (Zhang et al. 2006; Nam et al. 2006; Chung and Shin 2007; Hiemori et al. 2009). The concept of the total antioxidant capacity, which represents the ability of different food antioxidants to scavenge free radicals, has been suggested as a tool for evaluating the health effects of antioxidant rich foods. In non-pigmented rice varieties, the bran fraction has a total phenolic content (TPC) of 596.3 mg GAE 100 g^−1^, which is close to that of the husk (599.2 mg GAE 100 g^−1^) followed by the whole grain (263.9 mg GAE 100 g^−1^) and the rice endosperm (56.9 mg GAE 100 g^−1^) (Goufo and Trindade 2014). The phenolic compounds are mainly associated with the pericarp colour, darker the pericarp higher the amount of polyphenols (Tian et al. 2004; Zhou et al. 2004; Yawadio et al. 2007). Shen et al. (2009) characterized coloured parameters of rice grain (white, red and black rice) in wide collection of rice germplasm and found significantly associated with total phenolics, flavonoid and antioxidant capacity in three types of rice grain. Moreover, the correlations among the white rice accessions are rather weak. Goffman and Bergman (2004) evaluated different colour of rice genotypes and their total phenolic content ranged from 1.90 to 50.32 mg GAE g^−1^ of bran, and between 0.25 and 5.35 mg GAE g^−1^ of grain. Recent evidence of Goufo and Trindade (2014), showed 12 phenolic acids are generally identified in rice ranging from 177.6 to 319.8 mg 100 g^−1^ in the bran, 7.3 to 8.7 mg 100 g^−1^ in the endosperm, 20.8 to 78.3 mg 100 g^−1^ in the whole grain, and 477.6 mg 100 g^−1^ in the husk, depending on the rice color. This suggest that, rice bran has highest source of phenolic acids than others consumable part of rice. Numerous literatures have shown that consumption of colored rice reduces oxidative stress and simultaneously increases in antioxidant capacity. Consumption of colored rice varieties is very limited in Western countries, but in some growing areas of Asia, traditional varieties with colored pericarp are particularly valued in local markets (Finocchiaro et al. 2007).

The antioxidant compounds in rice as γ-oryzanols, tocols and phenolic acids associated with reduced risk of developing chronic diseases (Liu 2007; Yawadio et al. 2007). Among the various phenolic compounds, ferulic acid (56–77% of total phenolic acids) found in the endosperm, bran, and whole grain, followed by p-coumaric acid (8–24%), sinapic acid (2–12%), gallic acid (1–6%), protocatechuic acid (1–4%), p-hydroxybenzoic acid (1–2%), vanillic acid (1%), and syringic acid (1%) (Goufo and Trindade 2014).

### Effect of phytic acid in rice grain

An important mineral storage compound in seed is phytate, a mixed cation salt of phytic acid (InsP6) accounted approximately 75% of total phosphorus in seeds (Lott 1984; Suzuki et al. 2007; Raboy 2009). A considerable part of the phosphorus taken up by plants from soil is translocated ultimately to the seed and synthesized into phytic acid (PA). Therefore, this compound represents a major pool in the flux of phosphorus and recently estimated that, the amount of phosphorus synthesized into seed in the form of PA by crops each year represents a sum equivalent to >50% of phosphorus fertilizer used annually world-wide (Lott et al. 2000). Phytate being vital for seed development and higher seedling vigour, often considered as an anti-nutritional substance, but may have a positive nutritional role as an antioxidant, anti-cancer agent, lowering chronic disease rates, heart diseases in humans and prevents coronary diseases (Bohn et al. 2008; Gemede 2014). PA is considered as an anti-nutritional factor, as it forms complexes with proteins in seeds and essential minerals, such as Fe, Zn and Ca. (Reddy et al. 1996; Mendoza 2002; Bohn et al. 2008; Tamanna et al. 2013). However, Welch and Graham (2004) finding indicates that, PA have no much negative effects on Fe and Zn bioavailability.

## Prerequisite for improvement of Fe and Zn content in rice grain

Iron and zinc micronutrients are the most important elements, deficiency of which is a major cause for malnutrition. More than half of the world population is suffering from bioavailable nutrient deficiencies particularly in developing countries (Seshadri 1997; Shahzad et al. 2014). The main reason of these deficiency occurred due to consumption of polished cereal based food crops as rice, wheat and maize (Pfeiffer and McClafferty 2007). Modern high yielding rice varieties are poor sources of essential micronutrients like Fe and Zn (Zimmerman and Hurrel 2002). On an average, polished rice has 2 mg kg^−1^, while the recommended dietary intake of Fe for humans is 10–15 mg kg^−1^. Therefore, globally more than 3 billion people were affected by Fe deficiency, particularly in developing countries (Graham et al. 1999; Welch and Graham 2004). Pregnancy maternal mortality by anemia leads to 1.15 lakh deaths per year, resulting in 3.4 million disability-adjusted life-years (DALYs), has been recognized to Fe deficiency (Stoltzfus et al. 2004). Hence, improvement of Fe content in rice grain is necessary, which is a major challenge to the plant breeders. In plants, Zn plays a significant role in the biosyntheses and turnovers of proteins, nucleic acids, carbohydrates and lipids, with functional aspects as integral cofactor for more than 300 enzymes, coordinating ion in the DNA-binding domains of transcription factors and equally important as Fe and vitamin A (Marschner 1995). Males within the age bracket of 15–74 years require approximately 12–15 mg of Zn daily, while females within 15–74 years of age group need about 68 mg of Zn (Sandstead 1985). Generally, the content of Zn in polished rice is an average of only 12 mg kg^−1^, whereas the recommended dietary intake of Zn for humans is 12–15 mg kg^−1^ (FAO 2001). About 17.3% of the global population is under risk of Zn deficiency and in some regions of the world, it is as high as 30% due to dietary inadequacy (Wessells and Brown 2013). Therefore, to enhance the concentration of these micronutrients in rice grain could be possible as signified the presence of vast genetic potential of various rice germplasm by adapting appropriate genetic approaches (Fig. 1). However, major attention to date has been paid on identification and development of genetically engineered rice grains with increased bioavailable contents of Fe and/or Zn. The list of rice cultivars that possess dense micronutrient are presented in Table 1. Recently, Indian Institute of Rice Research, Hyderabad has developed a genotype (IET 23832) that possesses high Zn (19.50 ppm). As the brown rice has higher amount of Fe and Zn, more than 70% of micronutrients are lost during polishing (Sellappan et al. 2009) as they are located on the outer layer of the kernel. Martinez et al. (2010) found 10–11 ppm Fe and 20–25 ppm Zn in brown rice, while 2–3 ppm Fe and 16–17 ppm Zn was observed in milled rice.

**Table 1:** List of identified promising donors for Fe and Zn nutritional quality traits in rice

S. no	Rice genotypes	Nutritional element	Reference
1	SL-32, Annada, ASD16, CH-45, Nagina 22, Swarna, IR-29, Pusa Sugandha-1, IRGC-106187, IR68144-3B-2-2-3, IRGC-105320, IRGC-105320, IRGC-86476, CH-45, Jyoti, HKR-126, Varsha, MSE-9, Jalmagna, Zuchem, Kalabath, Pusa Basmati, Noothipattu, Pitchavari, Thanu, TKM-9, NDR-6279, and Aghonibora	Fe (>20 ppm)	Gregorio (2002), Anandan et al. (2011), Anuradha et al. (2012), Ravindra Babu (2013), Jagadeesh et al. (2013)
2	Nagina 22, Honduras, RG-187, SL-32, Aghoni bora, Annada, ASD-16, Jalmagna, CH-45, BPT-5204, Lalat, Sasyasri, Swarna, IR-29, Pusa Sugandha-1, IRGC-106187, IRGC-105320, IRGC-86476, Benibhog, CH-45, Jyoti, HKR-126, Pant Sugandh-17, Ratna, Chitiimutyalu, Ranbir basmati, IRRI-38, Jeerigesanna, Kalabath, Pusa Basmati, Noothipattu, Madhukar, Swarna, AM-141, Thanu, TKM-9, NDR-6279, Aghonibora and Pitchavari	Zn (>20 ppm)	Anandan et al. (2011), Anuradha et al. (2012), Ravindra Babu (2013), Berhanu et al. (2013) Jagadeesh et al. (2013), Vishnu et al. (2014), Gande et al. (2014)

## QTLs linked to nutritional and nutraceutical properties of rice

### QTLs for protein content in rice

Protein content in rice grain is a key factor for the enhancement of nutritional values and influencing the palatability of cooked rice (Matsue et al. 1995). Tan et al. (2001) mapped two QTLs for PC in the interval of C952-Wx on chromosome 6 near to waxy gene with 13% PV and LOD score of 6.8 and another QTL was mapped within the interval of R1245-RM234 on chromosome 7, which accounted for 4.7% of the PV and LOD score of 3.2. On the other hand, Aluko et al. (2004) identified four QTLs located on chromosomes 1, 2, 6 and 11 in a DH population from an inter specific crosses between *O. sativa* and *O. glaberrima*. Among the four QTLs, one QTL was located on chromosome 6, which is closely associated with *Wx* gene influencing rice quality.

Three QTLs viz., *qPC1.1, qPC11.1,* and *qPC11.2* were associated with PC of brown rice (Qin et al. 2009). Among them, *qPC11.1,* and *qPC11.2* were identified on chromosome 11 exhibiting 22.10% and 6.92% PV with LOD score of 4.90 and 2.75, respectively. The QTL *qPC11.2* was found to be consistent over two years of trial and linked with marker RM287. Yu et al. (2009) detected five QTLs for PC and four QTLs for fat content from 209 RILs. The five QTLs (*qPC*-*3, qPC*-*4, qPC*-*5, qPC*-*6* and *qPC*-*10*) for PC were detected on chromosomes 3, 4, 5, 6 and 10 with LOD score of 6.25, 2.87, 2.28, 9.78 and 4.50 respectively. Among these five loci, *qPC*-*6* observed to be nearer to the *Wx* marker between RM190 and RZ516 on the short arm of rice chromosome 6, explaining 19.3% of the PV and other four QTLs explained 3.9–10.5% of the PV. Zhong et al. (2011) reported two consistent QTLs for PC in milled rice as *qPr1* and *qPr7* detected over two years and positioned in the marker interval of RM493-RM562 and RM445-RM418 on chromosome 1 and 7 respectively. Recently, three QTLs *qPro*-*8*, *qPro*-*9* and *qPro*-*10* were detected on chromosome 8 flanked by RM506-RM1235 with a LOD score of 2.57, chromosome 9 in the interval of RM219-RM23914 with a LOD score of 2.66, and chromosome 10 separated by RM24934-RM25128 with a LOD score of 6.13 respectively for PC from 120 DH lines (Yun et al. 2014).

### QTLs associated with amino acid in rice

Amino acid (AA) composition and mapping was reported in milled rice using 190 RILs and detected eighteen chromosomal regions for 17 out of 20 AA (except Tryptophan, Glutamine, and Asparagine), essential AA in total and total AA content in rice grain (Wang et al. 2008). Two major QTL clusters in RM472-RM104 (1–19) and RM125-RM542 (7–4, 5) were detected consistently in two years and explained about 30 and 40% of PV. Zhong et al. (2011) detected 48 and 64 QTLs related to AA in the year of 2004 and 2005, respectively. Most QTLs co-localized, forming 29 QTL clusters on the chromosomes with three major ones detected in both years, which were mapped on chromosomes 1, 7 and 9, respectively. The two QTL clusters for amino acid content, *qAa1* and *qAa7*, influenced almost all the traits and the third QTL cluster for amino acid content, *qAa9*, increased the lysine content. Therefore, these identified QTLs and their association with particular grain quality nutrient trait results will be useful to find the candidate genes and favorable alleles to transfer into elite breeding rice cultivars through marker-assisted breeding program.

### QTLs responsible for mineral contents in rice

Several QTLs related to nutritional quality traits have been reported in rice from different genetic backgrounds of intraspecific and interspecific crosses using molecular markers. The grain nutrient traits associated with various QTLs and linked/flanking markers are summarized in Table 2 and Fig. 2. Three loci explaining 19–30% variation for Fe content on chromosomes 7, 8, and 9 were observed by Gregorio et al. (2000). A major QTL explaining 16.5% of PV for Fe content on chromosome 2 was identified from a DH population derived from a cross between IR64 and Azucena (Stangoulis et al. 2007). Besides, Garcia-Oliveira et al. (2008) reported a QTL for Fe content close to the marker RM6641 on chromosome 2 from an introgression line derived from a cross between Teqing and *Oryza rufipogon*. Wild rice (*O. rufipogon*) contributed favorable alleles for most of the QTLs (26 QTLs), and chromosomes 1, 9 and 12 exhibited 14 QTLs (45%) for these traits. One major effect of QTL for zinc content accounted for the largest proportion of phenotypic variation (11–19%) was detected near the simple sequence repeats marker RM152 on chromosome 8. James et al. (2007) used a DHs population for three Fe linked QTLs on chromosomes 2, 8 and 12, explaining 17, 18 and 14% of the total PV, respectively. They also reported two QTLs for Zn content on chromosomes 1 and 12, explaining PV of 15 and 13% respectively. Norton et al. (2010) reported ten QTLs for five mineral elements (Cu, Ca, Zn, Mn and Fe) and Fe (*qFe*-*1*) mineral trait explained the highest PV of 25.81% with LOD score of 7.66. Anuradha et al. (2012) identified 14 QTLs for Fe and Zn from unpolished rice of Madhukar/Swarna RILs. Seven QTLs each for grain Fe and Zn content were identified on chromosomes 1, 3, 5, 7 and 12 and the PV ranged from 29 to 71%. In addition, Gande et al. (2014) identified 24 candidate gene markers responsible for Zn content and four candidate genes namely *OsNAC, OsZIP8a, OsZIP8c* and *OsZIP4b* showed significant PV of 4.5, 19.0, 5.1 and 10.2%, respectively.

**Table 2:** List of rice nutrient traits associated with different QTLs (>3.0 LOD) mapped in different rice population

S. no	Grain traits	Chr	QTLs	Markers	Type	Peak marker	Populations	References
1	PC	1	*qPr1*	RM493-RM562	RILs	RM493-RM562	Zhenshan97B/Delong 208	Zhong et al. (2011)
2	PC	1	*qPC1.1*	1008-RM575	DHs		Samgang/Nagdong	Qin et al. (2009)
3	MAC-P	1	*qP.1*	RM3411	LT/TL-RILs		TeQing/Lemont	Zhang et al. (2014)
4	MAC-K	1	*qK.1*	RM5501	LT/TL-RILs		Lemont/TeQing	Zhang et al. 2014
5	PC	1	*qPC1*	RM472-RM104	RILs		Zhenshan97/Nanyangzhan	Peng et al. (2014)
6	AAC	1	*qAa1*	RM493-RM562	RILs		Zhenshan97B/Delong 208	Zhong et al. (2011)
7	MAC-P	1	*qP.1*	RM495	LT/TL-RILs		Lemont/TeQing	Zhang et al. (2014)
8	MAC-Cd	1	*qCd.1*	RM6840	LT-RILs			
9	Zn	1	*qZn.1*	RM34-RM237	DHs		IR64/Azucena	James et al. (2007)
10	Mn	1	*qMn.1*	RM243-RM312	DHs			
11	MAC-Co	1	*qCo.1*	RM490	LT/TL-RILs		Lemont/TeQing	Zhang et al. (2014)
12	MAC-Ca	1	*qCa1*-*1*	RM6480	ILs		*O. rufipogon*/Teqing	Garcia-Oliveira et al. (2008)
13	MAC-P	1	*qP1*-*1*	RM212	ILs			
14	Fe	1	*qFe1.1*	RM243-RM488	RILs		Madhukar/Swarna	Anuradha et al. (2012)
15	Fe	1	*qFe1.2*	RM488-RM490	RILs			
16	AAC-Asp/Thr/Glu/Gly/Ala/Cys/Tyr/Pro/Eaa/total	1	*qAA.1*	RM472-RM104	RILs	RM472	Zhenshan97/Nanyangzhan	Wang et al. (2008)
17	Fe	1	*qFe.1*	RM259-RM243	RILs	RM259-RM243	Zhenshan 97/Minghui 63	Kaiyang et al. (2008)
18	MIC-Fe	2	*qFe2*-*1*	RM6641	ILs		*O. rufipogon*/Teqing	Garcia-Oliveira et al. (2008)
19	PC	2	*qPC*-*2*	RM5897-RM6247	RILs		Chuan7/Nanyanghan	Lou et al. (2009)
20	MIC-Cu	2	*qCu.2*	RM6378	LT/TL-RILs		Lemont/TeQing	Zhang et al. (2014)
21	MAC-Sr	2	*qSr.2*	RM3688	LT-RILs			
22	Fe	2	*qFe.2*	RM53-RM300	DHs	RM53-RM300	IR64/Azucena	James et al. (2007)
23	AAC-His	2	*qAA.2*	RM324-RM301	RILs	RM301	Zhenshan97/Nanyangzhan	Wang et al. (2008)
24	AAC-Val/Ile/Leu/His/Phe	2	*qAA.2*	RM322-RM521	RILs	RM521		
25	PC	2	*qLip*-*2*	RM5619-RM1211	DHs		Cheongcheong/Nagdong	Yun et al. (2014)
26	PC	2	*qPro*-*2*	RM12532-RM555	DHs		Cheongcheong/Nagdong	Lee et al. (2014)
27	MIC-Fe	2	*qFe.2*	RM452	LT/TL-RILs		Lemont/TeQing	Zhang et al. (2014)
28	MIC-Mn	2	*qMn2*-*1*	RM6367	ILs		*O. rufipogon*/Teqing	Garcia-Oliveira et al. (2008)
29	MAC-S	2	*qS.2*	RM266	LT-RILs		Lemont/TeQing	Zhang et al. (2014)
30	MAC-Ca	3	*qCa.3*	RM5626-RM16	LT/TL-RILs			
31	MAC-Rb	3	*qRb.3*	RM489	LT-RILs			
32	AAC-Tyr	3	*qAA.3*	RM520-RM468	RILs	RM520	Zhenshan97/Nanyangzhan	Wang et al. (2008)
33	MAC-Mg	3	*qMg3*-*1*	RM5488	ILs		O*. rufipogon*/Teqing	Garcia-Oliveira et al. (2008)
34	Ca	3	*qCa.3.*	RM200-RM227	RILs		Zhenshan 97/Minghui 63	Kaiyang et al. (2008)
35	PC	3	*qPC*-*3*	RM251-RM282	RILs		Xieqingzao B/Milyang	Yu et al. (2009)
36	Zn	3	*qZn3.1*	RM7-RM517	RILs		Madhukar × Swarna	Anuradha et al. (2012)
37	PC	3	*qPC*-*3*	RM251-RM282	RILs		Xieqingzao B/Milyang	Yu et al. (2009)
38	Mn	3	*qMn.3*	RM227-R1925	RILs		Zhenshan 97/Minghui 63	Kaiyang et al. (2008)
39	Cu	3	*qCu.1*	R1925-RM148	RILs	R1925-RM148		
40	AAC-Thr/Gly/His/Arg	4	*qAA.4*	RM348-RM131	RILs	RM131	Zhenshan97/Nanyangzhan	Wang et al. (2008)
41	CPB	4	*qcpb4*	E12M61.256	RILs		Cypress/Panda	Kepiro et al. (2008)
42	CPH	4	*qcph4*	E12M61.256	RILs			
43	Cu	5	*qCu.5*	C1447-RM31	RILs		Zhenshan 97/Minghui 63	Kaiyang et al. (2008)
44	PA	5	*qPA.5*	RM305-RM178	DHs		IR64/Azucena	James et al. (2007)
45	FC	5	*qFC*-*5*	RG480-RM274	RILs		Xieqingzao B/Milyang	Yu et al. (2009)
46	Fe	5	*qFe5.1*	RM574-RM122	RILs		Madhukar/Swarna	Anuradha et al. (2012)
47	MAC-Ca	5	*qCa5*-*1*	RM598	ILs		*O. rufipogon*/Teqing	Garcia-Oliveira et al. (2008)
48	MIC-Zn	5		RM421	LT/TL-RILs		Lemont/TeQing	Zhang et al. (2014)
49	LC	6	*qLIp*-*6*	RM586-RM1163	DHs		Cheongcheong/Nagdong	Yun et al. (2014)
50	PC	6	*qPC*-*6*	RM190-RZ516	RILs	RM190-RZ516	Xieqingzao B/Milyang	Yu et al. (2009)
51	FC	6	*qFC*-*6*	RM190-RZ516	RILs	RM190-RZ516	Xieqingzao B/Milyang	Yu et al. (2009)
52	MIC-Cu	6	*qCu6*-*1*	RM204	ILs		O. rufipogon/Teqing	Garcia-Oliveira et al. (2008)
53	Zn	6	*qZn.6*	RZ398-RM204	RILs		Zhenshan 97/Minghui 63	Kaiyang et al. (2008)
54	PC	6	*qPC*-*6*	RM190-RZ516	RILs		Xieqingzao B/Milyang	Yu et al. (2009)
55	MAC-Mg	6	*qMg.6*	OSR 21	LT/TL-RILs		Lemont/TeQing	Zhang et al. (2014)
56	PC	7	*qPc7*	RM270-C751	DHs		Yuefu/IRAT109	Yongmei et al. (2007)
57	MIC-Mn	7	*qMn.7*	RM214	LT/TL-RILs		Lemont/TeQing	Zhang et al. (2014)
58	AAC-Pro/Gly/Met/Arg	7	*qAA.7*	RM125-RM214	RILs	RM214	Zhenshan97/Nanyangzhan	Wang et al. (2008)
59	Zn	7	*qZn7.3*	RM501-OsZip2	RILs		Madhukar/Swarna	Anuradha et al. (2012)
60	Fe	7	*qFe7.1*	RM234-RM248	RILs			
61	MAC-P	7	*qP.7*	RM70-RM172	DHs		IR64/Azucena	James et al. (2007)
62	PC	7	*qPC.1*	R1245-RM234	RILs		Zhenshan97/Minghui 63	Tan et al. (2001)
63	PC	7	*qPr7*	RM445-RM418	RILs		Zhenshan97B/Delong 208	Zhong et al. (2011)
64	MIC-Zn	8	*qZn8*-*1*	RM152	ILs		*O. rufipogon*/Teqing	Garcia-Oliveira et al. (2008)
65	AAC-Tyr	8	*qAA.8*	RM137-RM556	RILs	RM556	Zhenshan97/Nanyangzhan	Wang et al. (2008)
66	AAC-Cys	8	*qAA.8*	RM447-RM458	RILs	RM447		
67	MAC-K	8	*qK8*-*1*	RM3572	ILs		*O. rufipogon*/Teqing	Garcia-Oliveira et al. (2008)
68	Zn	8	*qZn.8*	RM25-R1629	RILs		Zhenshan 97/Minghui 63	Kaiyang et al. (2008)
69	Cu	8	*qCu.8*	RM201-C472	RILs			
70	Fe	8	*qFe.8*	RM137-RM325A	DHs		IR64/Azucena	James et al. (2007)
71	AAC	9	*qAa9*	RM328-RM107	RILs		Zhenshan97B/Delong 208	Zhong et al. (2011)
72	MAC-P	9	*qP9*-*1*	RM201	ILs		*O. rufipogon*/Teqing	Garcia-Oliveira et al. (2008)
73	MAC-Mg	10	*qMg.10*	RM467	LT-RILs		Lemont/TeQing	Zhang et al. (2014)
74	AAC-Cys/Leu/Ile/Phe	10	*qAA.10*	RM467-RM271	RILs	RM271	Zhenshan97/Nanyangzhan	Wang et al. (2008)
75	PC	10	*qPC*-*10*	RM184-RM3229B	RILs		Xieqingzao B/Milyang	Yu et al. (2009)
76	PC	10	*qPro*-*10*	RM24934-RM25128	DHs	RM24934	Cheongcheong/Nagdong	Yun et al. (2014)
77	MAC-Mg	11	*qMg.11*	RM332	LT/TL-RILs		Lemont/TeQing	Zhang et al. (2014)
78	MIC-Cu	11	*qCu.11*	RM167	LT-RILs			
79	PC	11	*qPC1.11*	1027-RM287	DHs	RM287	Samgang and Nagdong	Qin et al. (2009)
80	Fe	11	*qFe.11*	RZ536-TEL3	RILs		Zhenshan 97/Minghui 63	Kaiyang et al. (2008)
81	PC	11	*qPC1.11*	RM287-RM26755	DHs	RM287	Samgang and Nagdong	Qin et al. (2009)
82	PA	12	*qPA.12*	RM247-RM179	DHs		IR64/Azucena	James et al. (2007)
83	Fe	12	*qFe.12*	RM270-RM17	DHs			
84	Zn	12	*qZn.12*	RM235-RM17	DHs			
85	Fe	12	*qFe12.2*	RM260-RM7102	RILs		Madhukar/Swarna	Anuradha et al. (2012)
86	Fe	12	*qFe12.1*	RM17-RM260	RILs			
87	Zn	12	*qZn12.2*	RM260-RM7102	RILs			

*RB* rice bran (%), *NF* nutrition factors, *PC* protein content, *PA* phytic acid, *AAC* amino acid content, *CPB* crude protein brown rice, *CPH* crude protein head rice, *MIC* micro-element, *MAC* macro-element, *LC* lipid content, *FC* fat content

Garcia-Oliveira et al. (2008) identified 31 putative QTLs associated with microelements (Fe, Zn, Mn, Cu,) and macro elements (Ca, Mg, P and K) on all chromosomes except on chromosome 7. Among the total QTLs identified, chromosomes 1 and 9 had the highest number of QTLs having five QTLs each. Earlier reports showed several QTLs for the mineral content associated with different chromosomal regions of rice. QTLs for K on chromosomes 1 and 4 (Wu et al. 1998), P on chromosomes 1 and 12 (Ni et al. 1998; Wissuwa et al. 1998; Ming et al. 2001; Wissuwa and Ae 2001a, b) and Mn on chromosome 10 (Wang et al. 2002) were reported. Lu et al. (2008) observed 10 QTLs for Ca, Fe, Mn, and Zn accumulation in rice grains on seven chromosomes. Zhang et al. (2014) reported 134 QTLs for 16 elements in unmilled rice grain and among them, six were considered strongly associated and validated.

### QTLs for phenolic compounds in rice grain

The *Rc* locus regulates pigmentation of the rice bran layer, and selection for the *rc* allele (white pericarp) occurred during domestication of the crop. Two loci, *Rc* and *Rd* were found to be responsible for the formation of pericarp colour (Sweeney et al. 2006; Furukawa et al. 2007). *Rc* produces brown pericarp and seed coat, with *Rd* it develops red pericarp and seed coat, while *Rd* alone has no phenotype. Rc encodes a regulatory protein (*Basic Helix*-*Loop*-*Helix Protein*) that allows the accumulation of proanthocyanidins (Sweeney et al. 2006), while Rd encodes the enzyme DFR (*dehydroflavonolreductase*), which is involved in anthocyanin and proanthocyanidins pathway (Furukawa et al. 2007). Consequently, wild-type allele (*Rc*), the domestication allele (*rc*) and a mutant allele (*Rc*-*s*) were cloned and sequenced. The allele *rc* was found to be null with 14-bp deletion, responsible for frame shift mutation and a premature stop codon (Brooks et al. 2008). Through classical genetic approaches, Yoshimura et al. (1997) identified two loci, *Pb* (*Prp*-*b*) and *Pp* (*Prp*-*a*), located on chromosomes 4 and 1, respectively for the pericarp pigmentation with anthocyanin of black rice. Further, Wang and Shu (2007) mapped *Pb* gene responsible for purple pericarp on chromosome 4 and suggested that, the gene *Pb* may be a mutant of gene *Ra* caused by a two bases deletion (GT) within exon 7 of the *Ra*. Bres-Patry et al. (2001) identified two QTLs controlling rice pericarp and it was located on chromosomes 1 and 7. By association mapping Yafang et al. (2011) and Shao et al. (2011) reported that RM339 and RM316 were the common markers for antioxidant, flavonoids and phenolic content. *Ra* and *Rc* were main effect loci for pericarp color and phenolic compounds.

### Associated QTLs for phytic acid

In rice, phytic acid (PA) is a major source of P for support of seedling growth on P-deficient soil and important role of anti-nutritional factor. Liu et al. (2005) reported the amount of PA and protein content (PC) in 24 cultivars of rice and found to be no significant correlation between them. Among the cultivars, PA content ranged from 0.68% for Xiu217 to 1.03% for Huai9746, with a mean of 0.873%, and PC ranged between 6.45% for Xiu52 and 11.10% for K45, with a mean of 8.26%. The molecular mechanism and genetic trait of phytate accumulation in rice grain is necessary to understand for designing a breeding program. James et al. (2007) identified two QTLs for phytate concentration on chromosomes 5 and 12 with LOD score of 5.6 and 3.5 explaining 24.3 and 15.4% of PV, respectively. In addition, they reported significant positive correlation of phytate with inorganic P and total P (R = 0.99), indicating that the majority of P in grain was stored in the form of phytate.

## Achievements through transgenic approaches to enhance nutritional values

Genetic engineering, an alternative approach to enhance nutritional values, has been considered to be the potential tool for the sustainable and efficient strategy for increasing the nutritional quality traits in target area of plants (Uzogara 2000; Lucca et al. 2001; Zimmerman and Hurrel 2002; Dias and Ortiz 2012). The world population would likely to reach 8 billion by 2030. Therefore, the problem of malnutrition would further exaggerated to 93% (Khush 2005, 2008). Numerous evidences are piling up showing significant increase of bioavailable content in rice grains by transfer of biofortfication genes through biolistic and *Agrobacterium*-mediated transformation method (Table 3). Through the transgenic approaches, Goto et al. (1999) first observed 3-fold enhancement of Fe in the starchy endosperm of rice by transferring the *ferritin* gene of soybean. Similarly, in 2001 Lucca et al. introduced *ferritin* gene from common bean into rice showed 2-fold concentration of Fe in seeds as compared to controls. Vasconcelos et al. (2003) transferred soybean *ferritin* gene into rice and observed 3-fold increase of Fe in milled rice and 2-fold in rough rice. Similarly, Khalekuzzaman et al. (2006) observed increase in Fe T_1_ brown seeds and T_2_ polish rice seeds compared to control. Thus, the Fe content increased more than 2-fold in transgenic lines. Subsequently many researchers have attempted to increase Fe content in rice endosperm by over expressing genes involved in Fe uptake from the soil and translocation from root, shoot, flag leaf to grains, and by increasing the efficiency of Fe storage proteins (Kobayashi and Nishizawa 2012; Lee et al. 2012; Bashir et al. 2013; Masuda et al. 2013; Slamet-Loedin et al. 2015). Several studies exhibited the associated increase in Fe and Zn content in rice grain obtained by over expression or activation of the *Nicotianamine Synthase* (NAS) genes or influenced with other transporters genes (Table 3). Masuda et al. (2009) transferred *NAS* gene of *Hordeum vulgare* to rice observed significant enhancement of the target trait, which accumulated 2- to 3-fold higher iron and zinc in polished rice grain. Zheng et al. (2010) observed 5-fold iron accumulation in polished rice grain through the over expression of endosperm specific endogenous NAS gene. Through the higher expression of three rice NAS homologous proteins, (*OsNAS1, OsNAS2*, and *OsNAS3*), Johnson et al. (2011) observed 2-fold increase in Fe and Zn concentration in polished rice (Table 4). Similarly, Lee et al. (2009) observed transfer of NAS gene (*OsNAS3*-*D1*) increases the expression of Fe (2.9-fold), Zn (2.2-fold), and Cu (1.7-fold) compared to wild type grain at seedling stage. Soumitra et al. (2012) observed 7.8-fold increase of Fe content in a line 276-1-2 and six lines showed a 4.1 to 4.5-fold increment over control by over expression of *ferritin* gene. Masuda et al. (2013) introduced multiple genes viz., *OsSUT1* promoter-driven O*sYSL2*, *ferritin* gene under the control of endosperm-specific promoter, barley *IDS3* genome fragment and NAS over expression and observed significant increase in 1.4-fold, 2-fold, 6-fold, 3-fold of Fe concentration respectively as compared to polished rice seeds. These results suggest that, targeting multiple genes would be more successful in enhancing nutritional values of rice.

**Table 3:** Incorporation of various nutritional genes into rice cultivars through genetic engineering approaches

S. no	Nutrient	Gene	Increases to fold expression (compare to wild type/non-transformed)	References
1	Vit A	*Nppsy1, EucrtI*	1.6-fold	Ye et al. (2000)
2	Fe	*Osnas2*	4.2-fold	Johnson et al. (2011)
		*Gm ferritin, Af phytase,* and *Osnas1*	4 to 6.3-fold	Wirth et al. (2009)
		*Activation tagging of Osnas3*	2.9-fold	Lee et al. (2009)
3	Zn	*Activation tagging of Osnas2*	2.9-fold	Lee et al. (2011)
		*Osnas2*	2.2-fold	Johnson et al. (2011)
		*Gm ferritin, Af phytase,* and *Osnas1*	1.6-fold	Wirth et al. (2009)
4	Fe	*Ferritin gene*	4.4-fold Fe	Vasconcelos et al. (2003)
5	Fe and Zn	*Nicotianamine synthase* (*NAS*) *gene*	2.0-fold Fe and 3.0-fold Zn	Masuda et al. (2009)
6	Fe and Zn	*OsNAS1, OsNAS2,* and *OsNAS3*	2.0-fold Fe and Zn	Johnson et al. (2011)
7	Fe and Zn	*Barley genes*	1.40-fold Fe and 1.35-fold Zn	Masuda et al. (2008)
8	β-carotene content	*Daffodil phytoene synthase* and *Erwinia phytoene desaturase*	2.3-fold	Beyer et al. (2002), Paine et al. (2005)
9	Fe	*SoyferH1*	3.0-fold Fe	Goto et al. (1999)
10	Fe and Zn	*SoyFerH1*	3.0-fold Fe and 1.1-fold Zn	Qu et al. (2005)
11	Fe	*PyFerritin, rgMT*	2.0-fold	Lucca et al. (2002)
12	Fe and Zn	*OsIRO2*	2.8-fold Fe and 1.4-fold Zn	Ogo et al. (2011)
13	Fe and Zn	*OsYSL15*	1.1-fold Fe and 1.0-fold Zn	Lee et al. (2009)
14	Fe and Zn	*HvNAS1, HvNAS1, HvNAAT,* and *IDS3*	1.2-fold Fe and 1.4-fold Zn	Suzuki et al. (2008)
15	Fe and Zn	*OsNAS1*	1.0-fold Fe and 1.3-fold Zn	Zheng et al. (2010)
16	Fe and Zn	*SoyFerH1*	2.5-fold Fe and 1.5-fold Zn	Paul et al. (2014)
17	Fe and Zn	*OsNAS2*	3.0-fold Fe and 2.7-fold Zn	Lee et al. (2012)
18	Fe and Zn	*HvNAS1*	2.5-fold Fe and 1.5-fold Zn	Higuchi et al. (2001)
19	Fe	*OsYSL2*	4.4-fold Fe	Ishimaru et al. (2010)
20	Fe and Zn	*AtNAS1, Pvferritin,* and *Afphytase*	6.3-fold Fe and 1.6-fold Zn	Wirth et al. (2009)
21	Fe and Zn	*SoyFerH2, HvNAS1,* and *OsYSL2*	3.4-fold Fe and 1.3-fold Zn	Aung et al. (2013)
22	Fe and Zn	*SoyFerH2, HvNAS1, HvNAAT*-*A,* -*B* and *IDS3 genome fragments*	2.5-fold Fe and 1.4-fold Zn	Masuda et al. (2013)
23	Zn, Cu, and Ni	*OsNAS3*	2.1, 1.5, and 1.3-fold	Lee et al. (2009)
24	Fe and Zn	*OsNAS3*-*D1*	1.7-fold Fe in shoots, 1.6-fold in Fe roots and 2.0-fold Zn in shoots, 1.6-fold Zn in roots	Lee et al. (2009)
25	Fe	*Ferritine gene*	2.0-fold Fe	Khalekuzzaman et al. (2006)
26	Fe and Zn	*Osfer2*	2.09-fold Fe and 1.37-fold zinc	Soumitra et al. (2012)

**Table 4:** Utilization of micronutrient traits related genes in rice for the improvement

S. no	Gene	Functions	References
1	OsZIP1	Vascular bundles, Epidermis and mesophyll celss	Lee et al. (2010), Ishimaru et al. (2011)
2	OsZIP3	Vascular bundles, Epidermal cells in stem	Ishimaru et al. (2011)
3	OsZIP4	Meristem, Vascular bundles, Epidermis and mesophyll celss	Lee et al. (2010), Ishimaru et al. (2011)
4	OsNAS3	Vascular bundles, Epidermis	Lee et al. (2010), Ishimaru et al. (2011)
5	OsYSL15	Fe transporters	Masuda et al. (2013)
6	OsYSL2, OsNAAT1 and OsNAC	High grain Zn content	Chandel et al. (2011)
7	OsNAC, OsZIP8a, OsZIP8c and OsZIP4	grain zinc content	Gande et al. (2014)
8	OsZIP8	Leaf blade, root, stem, anther, ovary and embryo	Bashir et al. (2012)
9	OsNRAMP7	High grain Zn content	Chandel et al. (2011)
10	OsNRAMP75	Mid grain filling stage	
11	OsNAAT1	High grain zn content	Chandel et al. (2011)
12	OsVIT1	High grain zn content	Chandel et al. (2011)
13	OsAAP6	grain protein content and nutritional quality	Peng et al. (2014)
14	Osfer2	Increases of iron content in grain	Paul et al. (2012)
15	MOT1(*molybdenum transporter* 1)	grain molybdenum concentration	Norton et al. (2014)
16	COPT1 and COPT2 (*copper transport*)	grain copper concentration	Norton et al. (2014)
17	Lsi1(*arsenic transport*)	inter and extra cellular transporters of arsenic	Ma et al. (2008), Norton et al. (2014)

Rice lacks the ability to produce *β*-*carotene,* the precursor of Vitamin A. Ye et al. (2000) developed golden rice that yields 1.6–2.0 μg g^−1^ of β-carotene of dry rice which is very beneficial for retina (Vitamin A) to create visual pigment and ultimately leads decreasing of night blindness and particularly useful for people in developing countries. It was possible by introgression of major four genes *phytoene synthase, phytoene desaturase, β*-*carotene desaturase*, and *lycopene β*-*cyclase* into rice.

## Advanced genomic technologies

The ever-increasing demand for rice production with higher quality drives to the identification of superior and novel rice cultivars. To meet these challenges, plant breeders and biotechnologist together has to explore efficient breeding strategies that integrate genomic technologies by using available germplasm resources to a new revolution in the field of plant breeding for better understanding of genotype and its relationship with the phenotype, in particular for complex traits. Genomic approaches are particularly useful when working with complex traits having multigenic and environmental effects. In this new plant breeding era, genomics will be an essential aspect to develop more efficient nutritional rich rice cultivars for reducing human health problems relating to mineral nutrition (Perez-de-Castro et al. 2012).

Sequenced rice genome has provided new technologies and tools in functional genomics, transcriptomics and proteomics of important agronomic traits in rice. At present, trends in molecular biology are fully updated. Therefore, by availing the different molecular approaches as, whole genome sequencing of 3000 rice accessions (Li et al. 2014), Genome-wide association mapping (Huang et al. 2010; Zhao et al. 2011; Varshney et al. 2014; McCouch et al. 2016; Yano et al. 2016; Wang et al. 2016; Edzesi et al. 2016; Biscarini et al. 2016; Si et al. 2016); Whole Genome SNP Array (Hu et al. 2013; Yu et al. 2014; Singh et al. 2015; Malik et al. 2016), Genomic-based genotyping platforms and re-sequencing (Gao et al. 2013; Han and Huang 2013; Chen et al. 2013; Barabaschi et al. 2016; Guo et al. 2014; Xu and Bai 2015), Genome-guided RNA-seq (Loraine et al. 2013; Szczesniak et al. 2013; Biselli et al. 2015; Peng et al. 2016; Badoni et al. 2016), Map-based cloning approach (Salvi and Tuberosa 2005; Price 2006; Shomura et al. 2008; Zhang et al. 2013), Transcriptome profiling (Mochida and Shinozaki 2010; Chandel et al. 2011; Venu et al. 2011), Genomics approaches (Mochida and Shinozaki 2010; Swamy and Kumar 2013; Varshney et al. 2014; Spindel et al. 2015; Okazaki and Saito 2016) Sequencing-By-Synthesis (SBS) (Venu et al. 2011; Sun et al. 2015), Next generation sequencing (NGS) technologies (Uchida et al. 2011; Miyao et al. 2012; James et al. 2013; Guo et al. 2014; Wang et al. 2016; Matsumoto et al. 2016) and etc. could be strategically exploited to understand molecular mechanism and their relation between the genotypes and phenotypic traits.

In 2011, Zhao et al. genotyped 413 diverse accessions of *O. sativa* with 44,100 SNP and phenotyped them for 34 traits including grain quality parameters. Deep transcriptional analysis by MPSS and SBS brought out several differentially expressed genes that affect milling yield and eating quality trait in rice (Venu et al. 2011). The genes that expressed were identified to be involved in biosynthesis of starch, aspartate amino acid metabolism, seed maturation and storage proteins.

Peng et al. (2016) developed a stable variant line (YVB) having greatly improved grain quality traits using restriction-site associated DNA sequencing technology (RADseq) from a BC_1_F_5_ backcross population derived from an *indica* hybrid rice maintainer line V20B and YVB line. The YVB is a stable variant line derived from V20B by transferring the genomic DNA of *O. minuta* into V20B using SIM method (Zhao et al. 2005). The deep re-sequencing of genomes of both the parents V20B and YVB showed read coverage of 89.04 and 93.13% and depth of sequencing 41.26-fold and 87.54-fold respectively. A total of 322,656 homologous SNPs were identified between V20B and YVB. A total of 17 QTLs for rice grain quality were detected on chromosomes 3, 5, 6, 8, and 9 through genetic map analysis with PV ranging from 5.67 to 35.07%. Invention of SIM technology enabling introduction of exogenous DNA helped in creating a large number of new rice germplasm accessions and the variants were analyzed using molecular markers (Pena et al. 1987; Zhao et al. 2005).

## Conclusion

The nutritional value enrichment of rice grain is very much essential to reduce malnutrition of developing countries in the post green revolution era. The current gain in knowledge on the nutritional value related genes and QTLs will help into develop desired genotypes for the humankind. The availability of gene based markers and advanced tool will assist breeders to accumulate specific alleles of genes known to play a role in nutritional grain quality traits in rice. In recent years, significant achievement has been made in genetic studies on grain protein and amino acid content, vitamins and minerals, glycemic index value, phenolic and flavinoid compounds, phytic acid, zinc and iron content along with QTLs linked to these traits but needs more research for processing and curative properties. Recent release of high protein and zinc rich rice varieties in India gives the positive note on progressive move in crop improvement program in rice. The, transgenic approach will further strengthen to enrich grain nutrition to desired level rapidly. The recent development of genomic technologies may augment for improving the nutritional quality in rice when it goes hand in hand with breeding program.

## Abbreviations

GI: glycemic index
PA: phytic acid
MIC: micro-elements
MAC: macro-elements
PC: protein content
EAA: essential amino acids
RBP: rice bran protein
RAG: rapidly available glucose
AC: amylose content
GAE: galleic acid equivalent
TPC: total phenolic content
AA: amino acid
DH: double haploid
PV: phenotypic variance
RIL: recombinant inbred lines
SNP: single nucleotide polymorphism
SSR: simple sequence repeats
STS: sequence tagged site

## References

[CR1] Adeyeye EI, Arogundade LA, Akintayo ET, Aisida OA, Alao PA (2000). Calcium, zinc and phytate interrelationship in some foods of major consumption in Nigeria. Food Chem.

[CR2] Aluko G, Martinez C, Tohme J, Castano C, Bergman C, Oard JH (2004). QTL mapping of grain quality traits from the interspecific cross *Oryza sativa* X *O. glaberrima*. Theor Appl Genet.

[CR3] Anandan A, Rajiv G, Eswaran R, Prakash M (2011). Genotypic variation and relationships between quality traits and trace elements in traditional and improved rice (*Oryza sativa* L.) genotypes. J Food Sci.

[CR4] Angwara S, Nittaya L, Suraphichaya K, Rungarun S, Kongkiat K, Siam P (2014) Rapidly available glucose (RAG) and waxy haplotype as indicators for glycemic index in some lowland and upland thai rice varieties (*Oryza sativa* L.). In: The 26th annual meeting of the thai society for biotechnology and international conference, pp 1–6

[CR5] Anuradha K, Agarwal S, Rao YV, Rao K, Viraktamath B, Sarla N (2012). Mapping QTLs and candidate genes for iron and zinc concentrations in unpolished rice of Madhukar × Swarna RILs. Gene.

[CR6] Aung MS, Masuda H, Kobayashi T, Nakanishi H, Yamakawa T, Nishizawa NK (2013). Iron biofortification of Myanmar rice. Front Plant Sci.

[CR7] Badoni S, Das S, Sayal YK, Gopalakrishnan S, Singh AK, Rao AR, Agarwal P, Parida SK, Tyagi AK (2016). Genome-wide generation and use of informative intron-spanning and intron-length polymorphism markers for high-throughput genetic analysis in rice. Sci Rep.

[CR8] Banerjee S, Sharma D, Verulkar S, Chandel G (2010). Use of in silico and semi quantitative RT-PCR approaches to develop nutrient rich rice (*Oryza sativa* L.). Ind J Biotechnol.

[CR9] Bao JS, Corke H, Sun M (2006). Nucleotide diversity in starch synthase IIa and validation of single nucleotide polymorphisms in relation to starch gelatinization temperature and other physicochemical properties in rice (*Oryza sativa* L.). Theor Appl Genet.

[CR10] Barabaschi D, Tondelli A, Desiderio F, Volante A, Vaccino P, Vale G, Cattivelli L (2016). Next generation breeding. Plant Sci.

[CR11] Bashir K, Ishimaru Y, Nishizawa NK (2012). Molecular mechanisms of zinc uptake and translocation in rice. Plant Soil.

[CR12] Bashir K, Takahashi R, Nakanishi H, Nishizawa NK (2013). The road to micronutrient biofortification of rice: progress and prospects. Front Plant Sci.

[CR13] Berhanu DB, Rakhia S, Naveen GK, Kundur PJ, Shashidhar HE (2013). Estimation of genetic variability and correlation studies for grain zinc concentrations and yield related traits in selected rice (Oryza Sativa L.) genotypes. Asian J Exp Biol Sci.

[CR14] Bert CYC, Casiana MVC, Kenneth LM, Parminder SV, David JM (2008) Rice molecular breeding laboratories in the genomics era: current status and future considerations. Int J Plant Genomics Article ID 524847 10.1155/2008/524847 PMC2408710 18528527

[CR15] Beyer P, Al-Babili S, Ye X, Lucca P, Schaub P, Welsch R, Potrykus I (2002). Golden rice: introducing the b-carotene biosynthesis pathway into rice endosperm by genetic engineering to defeat vitamin A deficiency. J Nutr.

[CR16] Biscarini F, Cozzi P, Casella L, Riccardi P, Vattari A, Orasen G, Perrini R, Tacconi G, Tondelli A, Biselli C, Cattivelli L, Spindel J, McCouch S, Abbruscato P, Vale G, Piffanelli P, Greco R (2016). Genome-wide association study for traits related to plant and grain morphology, and root architecture in temperate rice accessions. PLoS ONE.

[CR17] Biselli C, Bagnaresi P, Cavalluzzo D, Urso S, Desiderio F, Orasen G, Gianinetti A, Righettini F, Gennaro M, Perrini R, Ben Hassen M, Sacchi GA, Cattivelli L, Vale G (2015). Deep sequencing transcriptional fingerprinting of rice kernels for dissecting grain quality traits. BMC Genom.

[CR18] Bohn L, Meyer AS, Rasmussen SK (2008). Phytate: impact on environment and human nutrition. A challenge for molecular breeding. J Zhejiang Univ Sci B.

[CR19] Brand-Miller J, Stockmann K, Atkinson F, Petocz P, Denyer G (2000). Glycemic index, postprandial glycemia, and the shape of the curve in healthy subjects: analysis of a database of more than 1000 foods. Am J Clin Nutr.

[CR20] Brand-Miller J, Petocz P, Hayne S, Colagiuri S (2003). Low glycemic index diets in the management of diabetes: a meta analysis of randomized controlled trials. Diabetes Care.

[CR21] Bres-Patry C, Lorieux M, Clement G, Bangratz M, Ghesquiere A (2001). Heredity and genetic mapping of domestication-related traits in a temperate japonica weedy rice. Theor Appl Genet.

[CR22] Brooks SA, Yan W, Jackson AK, Deren CW (2008). A natural mutation in rc reverts white-rice-pericarp to red and results in a new, dominant, wild-type allele: Rc-g. Theor Appl Genet.

[CR23] Cagampang G, Cruz L, Espiritu S, Santiago R, Juliano BO (1966). Studies on the extraction and composition of rice proteins. Cereal Chem.

[CR24] Chandel GP, Samuel M, Dubey M, Meena R (2011). In silico expression analysis of QTL specific candidate genes for grain micronutrient (Fe/Zn) content using ESTs and MPSS signature analysis in rice (*Oryza sativa* L.). J Plant Genet Transgenics.

[CR25] Chen MH, Bergman C, Pinson S, Fjellstrom R (2008). Waxy gene haplotypes: associations with apparent amylose content and the effect by the environment in an international rice germplasm collection. J Cereal Sci.

[CR26] Chen MH, Bergman CJ, Pinson SRM, Fjellstrom RG (2008). Waxy gene haplotypes: associations with pasting properties in an international rice germplasm collection. J Cereal Sci.

[CR27] Chen H, He H, Zhou F, Yu H, Deng XW (2013). Development of genomics-based genotyping platforms and their applications in rice breeding. Curr Opin Plant Biol.

[CR28] Cheng A, Ismail I, Osman M, Hashim H (2012). Simple and rapid molecular techniques for identification of amylose levels in rice varieties. Int J Mol Sci.

[CR29] Chung HS, Shin JC (2007). Characterization of antioxidant alkaloids and phenolic acids from anthocyanin-pigmented rice (*Oryza sativa* cv. Heugjinjubyeo). Food Chem.

[CR30] Coffman WR, Juliano BO (1987) Rice. In: Olson RA, Frey KJ (eds) Nutritional quality of cereal grains: genetic and agronomic improvement. Agronomy monograph no. 28, American Society of Agronomy, Madison, pp 101–131

[CR31] Courage HK (2010) White rice raises risk of type 2 diabetes. Observations Scientific American, Blog Network, 14

[CR32] Dias JS, Ortiz R (2012). Transgenic vegetable breeding for nutritional quality and health benefits. Food Nutr Sci.

[CR33] Edzesi WM, Dang X, Liang L, Liu E, Zaid IU, Hong D (2016). Genetic diversity and elite allele mining for grain traits in rice (*Oryza sativa* L.) by association mapping. Front Plant Sci.

[CR34] FAO (2009) The state of food insecurity in the world 2009. FAO available at ftp://ftp.fao.org/docrep/fao/012/i0876e/i0876e.pdf

[CR35] FAO/WHO (2001) Human vitamin and mineral requirements. Report of a joint FAO/WHO expert consultation Bangkok, Thailand. http://www.fao.org/docrep/004/y2809e/y2809e0m.htm

[CR36] Finocchiaro F, Ferrari B, Gianinetti A, Dallasta C, Galaverna G, Scazzina F, Pellegrini N (2007). Characterization of antioxidant compounds of red and white rice and changes in total antioxidant capacity during processing. Mol Nutr Food Res.

[CR37] Fitzgerald M, Concepcion J, Rahman S, Resurreccion A, Bird AR, Morell MK (2011). Identification of a major genetic determinant of glycaemic index in rice. Rice.

[CR38] Furukawa T, Maekawa M, Oki T, Suda I, Iida S, Shimada H, Takamure I, Kadowaki K (2007). The Rc and Rd genes are involved in proanthocyanidin synthesis in rice pericarp. Plant J.

[CR39] Gande NK, Kundur PJ, Soman R, Ambati R, Ashwathanarayana R, Bekele BD, Shashidhar HE (2014). Identification of putative candidate gene markers for grain zinc content using recombinant inbred lines (RIL) population of IRRI38 X Jeerigesanna. Afr J Biotechnol.

[CR40] Gao ZY, Zhao SC, He WM, Guo LB, Peng YL, Wang JJ, Guo XS, Zhang XM, Rao YC, Zhang C, Dong GJ, Zheng FY, Lu CX, Hu J, Zhou Q, Liu HJ, Wu HY, Xu J, Ni PX, Zeng DL, Liu DH, Tian P, Gong LH, Ye C, Zhang GH, Wang J, Tian FK, Xuea DW, Liaoe Y, Zhua L, Chene MS, Lie JY, Chenga SH, Zhang GY, Wang J, Qiana Q (2013). Dissecting yield-associated loci in super hybrid rice by resequencing recombinant inbred lines and improving parental genome sequences. Proc Natl Acad Sci USA.

[CR41] Garcia-Oliveira AL, Tan L, Fu Y, Sun C (2008). Genetic identification of quantitative trait loci for contents of mineral nutrients in rice grain. J Integr Plant Biol.

[CR42] Gearing ME (2015) Good as gold: Can golden rice and other biofortified crops prevent malnutrition? Science in the News, Harvard University. http://sitn.hms.harvard.edu/

[CR43] Gemede HM (2014). Potential health benefits and adverse effects associated with phytate in foods. Food Sci Qual Manag.

[CR44] Goffman FD, Bergman CJ (2004). Rice kernel phenolic content and its relationship with antiradical efficiency. J Sci Food Agric.

[CR45] Goto F, Yoshihara T, Shigemoto N, Toki S, Takaiwa F (1999). Iron fortification of the rice seed by the soybean ferritin gene. Nat Biotechnol.

[CR46] Goufo P, Trindade H (2014). Rice antioxidants: phenolic acids, flavonoids, anthocyanins, proanthocyanidins, tocopherols, tocotrienols, γ-oryzanol, and phytic acid. Food Sci Nutr.

[CR47] Graham R, Senadhira D, Beebe S, Iglesias C, Monasterio I (1999). Breeding for micronutrient density in edible portions of staple food crops:conventional approaches. Fields Crops Res.

[CR48] Gregorio GB (2002). Progress in breeding for trace minerals in staple crops. J Nutr.

[CR49] Gregorio GB, Senadhira D, Htut H, Graham RD (2000). Breeding for trace mineral density in rice. Food Nutr Bull.

[CR50] Guo L, Gao Z, Qian Q (2014). Application of resequencing to rice genomics, functional genomics and evolutionary analysis. Rice.

[CR51] Han B, Huang XH (2013). Sequencing-based genome-wide association study in rice. Curr Opin Plant Biol.

[CR52] Han SW, Chee KM, Cho SJ (2015). Nutritional quality of rice bran protein in comparison to animal and vegetable protein. Food Chem.

[CR01] Helm RM, Burks AW (1996). Hypoallergenicity of rice bran protein. Cereal Foods World.

[CR53] Hiemori M, Koh E, Mitchell AE (2009). Influence of cooking on anthocyanins in black rice (*Oryza sativa* L. *japonica* var. SBR). J Agric Food Chem.

[CR54] Higuchi K, Watanabe S, Takahashi M, Kawasaki S, Nakanishi H, Nishizawa NK, Mori S (2001). Nicotianamine synthase gene expression differs in barley and rice under Fe—deficient conditions. Plant J.

[CR55] Hu C, Zawistowski J, Ling W, Kitts DD (2003). Black rice (*Oryza sativa* L. *indica*) pigmented fraction suppresses both reactive oxygen species and nitric oxide in chemical and biological model systems. J Agric Food Chem.

[CR56] Hu W, Wen M, Han Z, Tan C, Xing Y (2013). Scanning QTLs for grain shape using a whole genome SNP array in rice. J Plant Biochem Physiol.

[CR57] Huang X, Wei X, Sang T, Zhao Q, Feng Q, Zhao Y, Li C, Zhu C, Lu T, Zhang Z, Li M, Fan D, Guo Y, Wang A, Wang L, Deng L, Li W, Lu Y, Weng Q, Liu K, Huang T, Zhou T, Jing Y, Li W, Lin Z, Buckler ES, Qian Q, Zhang QF, Li J, Han B (2010). Genome-wide association studies of 14 agronomic traits in rice landraces. Nat Genet.

[CR58] Iqbal S, Bhanger MI, Anwar F (2005). Antioxidant properties and components of some commercially available varieties of rice bran in Pakistan. Food Chem.

[CR02] Iqbal A, Khalil IA, Ateeq N, Khan MS (2006). Nutritional quality of important food legumes. Food Chem.

[CR59] Ishimaru Y, Masuda H, Bashir K, Inoue H, Tsukamoto T, Takahashi M, Nakanishi H, Aoki N, Hirose T, Ohsugi R, Nishizawa NK (2010). Rice metal-nicotianamine transporter, OsYSL2, is required for the long-distance transport of iron and manganese. Plant J.

[CR60] Ishimaru Y, Bashir K, Nishizawa NK (2011). Zn uptake and translocation in rice plants. Rice.

[CR61] Jagadeesh BR, Krishnamurthy R, Surekha K, Yogesh GS (2013). Studies on high accumulation of iron and zinc contents in some selected rice genotypes. Glob J Biol Biotechnol.

[CR62] James CR, Huynh BL, Welch RM, Choi EY, Graham RD (2007). Quantitative trait loci for phytate in rice grain and their relationship with grain micronutrient content. Euphytica.

[CR63] James GV, Patel V, Nordstrom KJ, Klasen JR, Salome PA, Weigel D, Schneeberger K (2013). User guide for mapping-by-sequencing in Arabidopsis. Genome Biol.

[CR64] Johnson AA, Kyriacou B, Callahan DL, Carruthers L, Stangoulis J, Lombi E, Tester M (2011). Constitutive over expression of the OsNAS gene family reveals single gene strategies for effective iron- and zinc-biofortification of rice endosperm. PLoS ONE.

[CR65] Juliano BO, Bautista GM, Lugay JC, Reyes AC (1964). Studies on physico-chemical properties of rice. J Agric Food Chem.

[CR66] Kaiyang Lu, Li Lanzhi, Zheng Xingfei, Zhang Zhihong, Mou Tongmin, Zhongli Hu (2008). Quantitative trait loci controlling Cu, Ca, Zn, Mn and Fe content in rice grains. J Genet.

[CR67] Kawakatsu T, Yamamoto MP, Hirose S, Yano M, Takaiwa F (2008). Characterization of a new rice glutelin gene GluD-1 expressed in the starchy endosperm. J Exp Bot.

[CR68] Kepiro JL, McClung AM, Chen MH, Yeater KM, Fjellstrom RG (2008). Mapping QTLs for milling yield and grain characteristics in a tropical *japonica* long grain cross. J Cereal Sci.

[CR69] Khalekuzzaman M, Datta K, Olival N, Alam MF, Joarder I, Datta SK (2006). Stable integration, expression and inheritance of the ferritin gene intrans-genic elite *indica* rice cultivar BR29 with enhanced iron level in the endosperm. Indian J.

[CR70] Kharabian-Masouleh A, Waters DLE, Reinke RF, Ward R, Henry RJ (2012). SNP in starch biosynthesis genes associated with nutritional and functional properties of rice. Sci Rep.

[CR71] Khush GS (2005). What it will take to feed 5.0 billion rice consumers by 2030. Plant Mol Biol.

[CR72] Khush GS (2008). Biofortification of crops for reducing malnutrition. Proc Indian Natl Sci Acad.

[CR73] Kobayashi T, Nishizawa NK (2012). Iron uptake, translocation, and regulation in higher plants. Annu Rev Plant Biol.

[CR74] Kong J, Chia L, Goh N, Chia T, Brouillard R (2003). Analysis and biological activities of anthocyanins. Phytochemistry.

[CR75] Larkin PD, Park WD (2003). Association of waxy gene single nucleotide polymorphisms with starch characteristics in rice (*Oryza sativa* L.). Mol Breed.

[CR76] Lee S, Jeon US, Lee SJ, Kim YK, Persson DP, Husted S, Schjorring JK, Kakei Y, Masuda H, Nishizawa NK, Ana G (2009). Iron fortification of rice seeds through activation of the nicotianamine synthase gene. Proc Natl Acad Sci USA.

[CR77] Lee S, Jeong HJ, Kim SA, Lee J, Guerinot ML, An G (2010). OsZIP5 is a plasma membrane zinc transporter in rice. Plant Mol Biol.

[CR78] Lee S, Persson DP, Hansen TH, Husted S, Schjoerring JK, Kim YS, Jeon US, Kim YK, Kakei Y, Masuda H, Nishizhawa NK, An G (2011). Bio-available zinc in rice seeds is increased by activation tagging of nicotianamine synthase. Plant Biotechnol J.

[CR79] Lee S, Jeon JS, An G (2012). Iron homeostasis and fortification in rice. J Plant Biol.

[CR80] Lee GH, Yun BW, Kim KM (2014) Analysis of QTLs associated with the rice quality related gene by double haploid populations. Int J Genomics Article ID 781832 10.1155/2014/781832 PMC4247976 25478566

[CR81] Li JY, Wang J, Zeigler RS (2014). The 3000 rice genomes project: new opportunities and challenges for future rice research. Gigascience.

[CR82] Ling WH, Cheng QX, Ma J, Wang T (2001). Red and black rice decrease atheroscletoric plaque formation and increase antioxidant status in rabbits. J Nutr.

[CR83] Liu Q, Cui SW (2005). Understanding starches and their role in foods. Food carbohydrates: chemistry, physical properties and applications.

[CR84] Liu RH (2007). Whole grain phytochemicals and health. J Cereal Sci.

[CR85] Liu Z, Cheng F, Zhang G (2005). Grain phytic acid content in japonica rice as affected by cultivar and environment and its relation to protein content. Food Chem.

[CR86] Loraine AE, McCormick S, Estrada A, Patel K, Qin P (2013). RNA-seq of Arabidopsis pollen uncovers novel transcription and alternative splicing. Plant Physiol.

[CR87] Lott JNA, Murray DR (1984). Accumulation of seed reserves of phosphorus and other minerals. Seed physiology, vol 1, development.

[CR88] Lott JNA, Ockenden I, Raboy V, Batten GD (2000). Phytic acid and phosphorus in crop seeds and fruits: a global estimate. Seed Sci Res.

[CR89] Lou J, Chen L, Yue G, Lou Q, Mei H, Xiong L, Luo L (2009). QTL mapping of grain quality traits in rice. J Cereal Sci.

[CR90] Lu K, Li L, Zheng X, Zhang Z, Mou T, Hu Z (2008). Quantitative trait loci controlling Cu, Ca, Zn, Mn and Fe content in rice grains. J Genet.

[CR91] Lucca P, Hurrel R, Potrykus I (2001). Genetic engineering approaches to improve the bioavailability and the level of iron in the rice grains. Theor Appl Genet.

[CR92] Lucca P, Hurrell R, Potrykus I (2002). Fighting iron deficiency anemia with iron-rich rice. J Am Coll Nutr.

[CR93] Ma JF, Yamaji N, Mitani N, Xu XY, Su YH, McGrath SP, Zhao FJ (2008). Transporters of arsenite in rice and their role in arsenic accumulation in rice grain. Proc Natl Acad Sci.

[CR94] Malik N, Dwivedi N, Singh AK, Parida SK, Agarwal P, Thakur JK, Tyagi AK (2016). An integrated genomic strategy delineates candidate mediator genes regulating grain size and weight in rice. Sci Rep.

[CR95] Mammadov J, Aggarwal R, Buyyarapu R, Kumpatla S (2012) SNP markers and their impact on plant breeding. Int J Plant Genomics 12:Article ID 728398 10.1155/2012/728398 PMC3536327 23316221

[CR96] Manay NS, Shadaksharaswamy M (2001). Food facts and principles.

[CR97] Marschner H (1995). Function of mineral nutrients: micronutrients. Mineral nutrition of higher plants.

[CR98] Martinez CP, Borrero J, Taboada R, Viana JL, Neves P, Narvaez L, Puldon V, Adames A, Vargas A (2010) Rice cultivars with enhanced iron and zinc content to improve human nutrition. In: 28th International rice research conference, 8–12 November 2010, Hanoi, Vietnam OP10: Quality Grain, Health, and Nutrition

[CR99] Masuda H, Suzuki M, Morikawa KC, Kobayashi T, Nakanishi H, Takahashi M, Satoshi Mori MS, Nishizawa NK (2008). Increase in iron and zinc concentrations in rice grains via the introduction of barley genes involved in phytosiderophore synthesis. Rice.

[CR100] Masuda H, Usuda K, Kobayashi T, Ishimaru Y, Kakei Y, Takahashi M, Higuchi K, Nakanishi H, Mori S, Nishizawa NK (2009). Over expression of the barley nicotianamine synthase gene HvNAS1 increases iron and zinc concentrations in rice grains. Rice.

[CR101] Masuda H, Kobayashi T, Ishimaru Y, Takahashi M, Aung MS, Nakanishi H, Mori S, Nishizawa NK (2013). Iron-biofortification in rice by the introduction of three barley genes participated in mugineic acid biosynthesis with soybean ferritin gene. Front Plant Sci.

[CR102] Matsue Y, Odahara K, Hiramatsu M (1995). Differences in amylose content, amylographic characteristics and storage proteins of grains on primary and secondary rachis branches in rice (*Oryza Sativa*). Jpn J Crop Sci.

[CR103] Matsumoto T, Wu J, Itoh T, Numa H, Antonio B, Sasaki T (2016). The Nipponbare genome and the next-generation of rice genomics research in Japan. Rice.

[CR104] McCouch SR, Wright MH, Tung CW, Maron LG, McNally KL, Fitzgerald M, Singh N, DeClerck G, Agosto-Perez F, Korniliev P, Greenberg AJ, Naredo MEB, Mercado SMQ, Harrington SE, Shi Y, Branchini DA, Kuser-Falcao PR, Leung H, Ebana K, Yano M, Eizenga G, McClung A, Mezey J (2016). Open access resources for genome-wide association mapping in rice. Nat Commun.

[CR105] Mendoza C (2002). Effect of genetically modified low phyticacid plants on mineral absorption. Int J Food Sci Technol.

[CR106] Meng F, Wei Y, Yang X (2005). Iron content and bioavailability in rice. J Trace Elem Med Biol.

[CR107] Ming F, Zheng X, Mi G, Zhu L, Zhang F (2001). Detection and verification of quantitative trait loci affecting tolerance to low phosphorus in rice. J Plant Nutr.

[CR108] Misra A, Singhal N, Khurana L (2010). Obesity, the metabolic syndrome, and type 2 diabetes in developing countries: role of dietary fats and oils. J Am Coll Nutr.

[CR109] Miyao A, Nakagome M, Ohnuma T, Yamagata H, Kanamori H, Katayose Y, Takahashi A, Matsumoto T, Hirochika H (2012). Molecular spectrum of somaclonal variation in regenerated rice revealed by whole-genome sequencing. Plant Cell Physiol.

[CR110] Mochida K, Shinozaki K (2010). Genomics and bioinformatics resources for crop improvement. Plant Cell Physiol.

[CR111] Mohanty A, Marndi BC, Sharma S, Das A (2011). Biochemical characterization of two high protein rice cultivars from Assam rice collections. Oryza.

[CR112] Nagesh P, Usharani G, Neeraja N, Ravindra BV, Dayakar RT (2013). Molecular mapping of high iron and zinc rich regions in rice (*Oryza sativa* L.) grains using microsatellite markers. Helix.

[CR113] Nam SH, Choi SP, Kang MY, Koh HJ, Kozukue N, Friedman M (2006). Antioxidative activities of bran extracts from twenty one pigmented rice cultivars. Food Chem.

[CR114] Ni JJ, Wu P, Senadhira D, Huang N (1998). Mapping QTLs for phosphorus deficiency tolerance in rice (*Oryza sativa* L.). Theor Appl Genet.

[CR115] Norton GJ, Deacon CM, Xiong L, Huang S, Meharg AA, Price AH (2010). Genetic mapping of the rice ionome in leaves and grain: identification of QTLs for 17 elements including arsenic, cadmium, iron and selenium. Plant Soil.

[CR116] Norton GJ, Douglas A, Lahner B, Yakubova E, Guerinot ML, Pinson SR, Tarpley L, Eizenga GC, McGrath SP, Zhao FJ, Islam MR, Islam S, Duan G, Zhu Y, Salt DE, Meharg AA, Price AH (2014). Genome wide association mapping of grain arsenic, copper, molybdenum and zinc in rice (*Oryza sativa* L.) grown at four international field sites. PLoS ONE.

[CR117] Ogo Y, Itai RN, Kobayashi T, Aung MS, Nakanishi H, Nishizawa NK (2011). OsIRO2 is responsible for iron utilization in rice and improves growth and yield in calcareous soil. Plant Mol Biol.

[CR118] Ohstubo K, Nakamura S, Imamura T (2002). Development of the primer sets for identification of a rice variety, Koshihikari, by PCR. Nippon Nogeik Kaishi.

[CR119] Okazaki Y, Saito K (2016). Integrated metabolomics and phytochemical genomics approaches for studies on rice. GigaScience.

[CR120] Paine JA, Shipton CA, Chaggar S, Howells RM, Kennedy MJ, Vernon G, Wright SY, Hinchliffe E, Adams JL, Silverstone AL, Drake R (2005). A new version of golden rice with increased provitamin A content. Nat Biotechnol.

[CR121] Paul S, Ali N, Gayen D, Datta SK, Datta K (2012). Molecular breeding of Osfer2 gene to increase iron nutrition in rice grain. GM Crops Food.

[CR122] Paul S, Ali N, Datta SK, Datta K (2014). Development of an iron-enriched high-yieldings *indica* rice cultivar by introgression of a high-iron trait from transgenic iron-biofortified rice. Plant Foods Hum Nutr.

[CR03] Pena DL, Lorz H, Schell J (1987). Transgenic rye plant obtained by injecting DNA into young floral tillers. Nature.

[CR123] Peng B, Wang L, Fan C, Jiang G, Luo L, Li Y, He Y (2014). Comparative mapping of chalkiness components in rice using five populations across two environments. BMC Genet.

[CR124] Peng Y, Hu Y, Mao B, Xiang H, Shao Y, Pan Y, Sheng X, Li Y, Ni X, Xia Y, Zhang G, Yuan L, Quan Z, Zhao B (2016). Genetic analysis for rice grain quality traits in the YVB stable variant line using RAD-seq. Mol Genet Genomics.

[CR125] Perez-de-Castro AM, Vilanova S, Canizares J, Pascual L, Blanca JM, Diez MJ, Prohens J, Pico B (2012). Application of genomic tools in plant breeding. Curr Genomics.

[CR126] Pfeiffer WH, McClafferty B, Kang MS (2007). Biofortification: breeding micronutrient-dense crops. Breeding major food staples.

[CR127] Pingali PL, Hossain M, Gerpacio RV (1997). Asian rice bowls: the returning crisis?.

[CR128] Price A (2006). Believe it or not, QTLs are accurate!. Trends Plant Sci.

[CR129] Qin Y, Kim SM, Sohn JK (2009). QTL analysis of protein content in double-haploid lines of rice. Korean J Crop Sci.

[CR130] Qu LQ, Yoshihara T, Ooyama A, Goto F, Takaiwa F (2005). Iron accumulation does not parallel the high expression level of ferritin in transgenic rice seeds. Planta.

[CR131] Raboy V (2009). Approaches and challenges to engineering seed phytate and total phosphorus. Plant Sci.

[CR132] Ravindra Babu V (2013). Importance and advantages of rice biofortification with iron and zinc. An Open Access Journal published by ICRISAT. SAT eJ.

[CR133] Reddy MB, Hurrell RF, Juillerat MA, Cook JD (1996). The influence of different protein sources on phytate inhibition of nonheme-iron absorption in humans. Am J Clin Nutr.

[CR134] Renuka R, Arumughan C (2007). Phytochemical characterization of defatted rice bran and optimization of a process for their extraction and enrichment. Bioresour Technol.

[CR135] NRRI Annual Report (2014–2015) ICAR-National Rice Research Institute (NRRI), Cuttack

[CR136] Salvi S, Tuberosa R (2005). To clone or not to clone plant QTLs: present and future challenges. Trends Plant Sci.

[CR137] Sandstead HH (1985). Requirements of zinc in human subjects. J Am Coll Nutr.

[CR138] Saunders RM (1990). The properties of rice bran as a food stuff. Cereal Foods World.

[CR139] Schramm R, Abadie A, Hua N, Xu Z, Lima M (2007). Fractionation of the rice bran layer and quantification of vitamin E, oryzanol, protein, and rice bran saccharide. J Biol Eng.

[CR140] Sellappan K, Datta K, Parkhi V, Datta SK (2009). Rice caryopsis structure in relation to distribution of micronutrients (iron, zinc, b-carotene) of rice cultivars including transgenic *indica* rice. Plant Sci.

[CR141] Seshadri S (1997) Nutritional anemia in South Asia. In: Gillespie S (ed) Malnutrition in south asia—a regional profile. In: UNICEF Regional Office for South Asia, pp 75–124

[CR142] Shahzad Z, Rouached H, Rakha A (2014). Combating mineral malnutrition through iron and zinc biofortification of cereals. Compr Rev Food Sci Food Saf.

[CR143] Shao Y, Jin L, Zhang G, Lu Y, Shen Y, Bao J (2011). Association mapping of grain color, phenolic content, flavonoid content and antioxidant capacity in dehulled rice. Theor Appl Genet.

[CR144] Shen Y, Jin L, Xiao P, Lu Y, Bao JS (2009). Total phenolics, flavonoids, antioxidant capacity in rice grain and their relations to grain color, size and weight. J Cereal Sci.

[CR145] Shobha Rani N, Pandey MK, Prasad GSV, Sudharshan I (2006). Historical significance, grain quality features and precision breeding for improvement of export quality basmati varieties in India. Indian J Crop Sci.

[CR146] Shoji Y, Mita T, Isemura M, Mega T, Hase S, Isemura S, Aoyagi Y (2001). A fibronectin-binding protein from rice bran with cell adhesion activity for animal tumor cells. Biosci Biotechnol Biochem.

[CR147] Shomura A, Izawa T, Ebana K, Ebitani T, Kanegae H, Konishi S, Yano M (2008). Deletion in a gene associated with grain size increased yields during rice domestication. Nat Genet.

[CR148] Si L, Chen J, Huang X, Gong H, Luo J, Hou Q, Zhou T, Lu T, Zhu J, Shangguan Y, Chen E, Gong C, Zhao Q, Jing Y, Zhao Y, Li Y, Cui L, Fan D, Lu Y, Weng Q, Wang Y, Zhan Q, Liu K, Wei X, An K, An G, Han B (2016). OsSPL13 controls grain size in cultivated rice. Nat Genet.

[CR149] Singh N, Jayaswal PK, Panda K, Mandal P, Kumar V, Singh B, Mishra S, Singh Y, Singh R, Rai V, Gupta A, Raj Sharma T, Singh NK (2015). Single-copy gene based 50 K SNP chip for genetic studies and molecular breeding in rice. Sci Rep.

[CR150] Slamet-Loedin IH, Johnson-Beebout SE, Impa S, Tsakirpaloglou N (2015). Enriching rice with Zn and Fe while minimizing Cd risk. Front Plant Sci.

[CR151] Soumitra P, Nusrat A, Dipak G, Swapan KD, Karabi D (2012). Molecular breeding of Osfer2 gene to increase iron nutrition in rice grain. GM Crops Food Biotechnol Agric Food Chain.

[CR152] Spindel J, Begum H, Akdemir D, Virk P, Collard B, Redoña E, Atlin G, Jannink JL, McCouch SR (2015). Correction: genomic selection and association mapping in rice (*Oryza sativa*): effect of trait genetic architecture, training population composition, marker number and statistical model on accuracy of rice genomic selection in elite, tropical rice breeding lines. PLoS Genet.

[CR153] Srinivasa D, Raman A, Meena P, Chitale G, Marwaha A, Jainani KJ (2013). Glycaemic index (GI) of an Indian branded thermally treated basmati rice variety: a multi centric study. J Assoc Phys India.

[CR154] Stangoulis JCR, Huynh BL, Welch RM, Choi EY, Graham RD (2007). Quantitative trait loci for phytate in rice grain and their relationship with grain micronutrient content. Euphytica.

[CR155] Stoltzfus RJ, Mullany L, Black RE, Ezzati M, Lopez AD, Rodgers A, Murray CLJ (2004). Iron deficiency anaemia. Comparative quantification of health risks: global and regional burden of disease attributable to selected major risk factors.

[CR04] Sun SSM, Singh BK (1999). Methionine enhancement in plants. Plant amino acids: biochemistry and biotechnology.

[CR156] Sun H, Peng T, Zhao Y, Du Y, Zhang J, Li J, Xin Z, Zhao Q (2015). Dynamic analysis of gene expression in rice superior and inferior grains by RNA-seq. PLoS ONE.

[CR157] Suzuki M, Tanaka K, Kuwano M, Yoshida KT (2007). Expression pattern of inositol phosphate-related enzymes in rice (*Oryza sativa* L.): implications for the phytic acid biosynthetic pathway. Gene.

[CR158] Suzuki M, Morikawa KC, Nakanishi H, Takahashi M, Saigusa M, Mori S, Nishizawa NK (2008). Transgenic rice lines that include barley genes have increased tolerance to low iron availability in a calcareous paddy soil. Soil Sci Plant Nutr.

[CR159] Swamy BPM, Kumar A (2013). Genomics-based precision breeding approaches to improve drought tolerance in rice. Biotechnol Adv.

[CR160] Sweeney MT, Thomson MJ, Pfeil BE, McCouch SR (2006). Caught red-handed: Rc encodes a basic helix-loop-helix protein conditioning red pericarp in rice. Plant Cell.

[CR161] Szczesniak MW, Kabza M, Pokrzywa R, Gudys A, Makalowska I (2013). ERISdb: a database of plant splice sites and splicing signals. Plant Cell Physiol.

[CR162] Tamanna S, Sayma P, Sanjay K, Alak KD, Aysha FM, Ali S, Sunil KB, Zakir M, Howlader H (2013). Content of some minerals and their bioavailability in selected popular rice varieties from Bangladesh. Int J Curr Microbiol Appl Sci.

[CR163] Tan YF, Sun M, Xing YZ, Hua JP, Sun XL, Zhang QF, Corke H (2001). Mapping quantitative trait loci for milling quality, protein content and color characteristics of rice using a recombinant inbred line population derived from an elite rice hybrid. Theor Appl Genet.

[CR164] Tian S, Nakamura K, Kayahara H (2004). Analysis of phenolic compounds in white rice, brown rice, and germinated brown rice. J Agric Food Chem.

[CR165] Uchida N, Sakamoto T, Kurata T, Tasaka M (2011). Identification of EMS-induced causal mutations in a non-reference *Arabidopsis thaliana* accession by whole genome sequencing. Plant Cell Physiol.

[CR166] Uzogara SG (2000). The impact of genetic modification of human foods in the 21st century: a review. Biotechnol Adv.

[CR167] Varshney RK, Terauchi R, McCouch SR (2014). Harvesting the promising fruits of genomics: applying genome sequencing technologies to crop breeding. PLoS Biol.

[CR168] Vasconcelos M, Datta K, Oliva N, Khalekuzzaman M, Torrizo L, Krishnan S, Oliveirac M, Gotod F, Datta SK (2003). Enhanced iron and zinc accumulation in transgenic rice with the ferritin gene. Plant Sci.

[CR169] Venu RC, Sreerekha MV, Nobuta K, Belo A, Ning Y, An G, Meyers BC, Wang GL (2011). Deep sequencing reveals the complex and coordinated transcriptional regulation of genes related to grain quality in rice cultivars. BMC Genomics.

[CR170] Vishnu VN, Robin S, Sudhakar D, Rajeswari S, Raveendran M, Subramanian KS, Tannidi S, Balaji A (2014). Genotypic variation for micronutrient content in traditional and improved rice lines and its role in biofortification programme. Indian J Sci Technol.

[CR171] Wang CX, Shu QY (2007). Fine mapping and candidate gene analysis of purple peri-carp gene Pb in rice (*Oryza sativa* L.). Chin Sci Bull.

[CR172] Wang YX, Wu P, Wu YR, Yan XL (2002). Molecular marker analysis of manganese toxicity tolerance in rice under greenhouse conditions. Plant Soil.

[CR173] Wang L, Zhong M, Li X, Yuan D, Xu Y, Liu H, He Y, Luo L, Zhang Q (2008). The QTL controlling amino acid content in grains of rice (*Oryza sativa* L.) are co-localized with the regions involved in the amino acid metabolism pathway. Mol Breed.

[CR174] Wang H, Xu X, Vieira FG, Xiao Y, Li Z, Wang J, Nielsen R, Chu C (2016). The power of inbreeding: NGS-based GWAS of rice reveals convergent evolution during rice domestication. Mol Plant.

[CR175] Welch R, Graham RD (1999). A new paradigm for world agriculture: meeting human needs. Productive, sustainable, nutritious. Field Crops Res.

[CR176] Welch RM, Graham RD (2004). Breeding for micronutrients in staple food crops from a human nutrition perspective. J Exp Bot.

[CR177] Weng J, Gu S, Wan X, Gao H, Guo T, Su N, Lei C, Zhang X, Cheng Z, Guo X, Wang J, Jiang L, Zhai H, Wan J (2008). Isolation and initial characterization of GW5, a major QTL associated with rice grain width and weight. Cell Res.

[CR178] Wessells KR, Brown KH (2013). Estimating the global prevalence of zinc deficiency: results based on zinc availability in national food supplies and the prevalence of stunting. PLoS ONE.

[CR179] Wirth J, Poletti S, Aeschlimann B, Yakandawala N, Drosse B, Osorio S, Tohge T, Fernie AR, Gunther D, Gruissem W, Sautter C (2009). Rice endosperm iron biofortification by targeted and synergistic action of nicotianamine synthase and ferritin. Plant Biotechnol J.

[CR180] Wissuwa M, Ae N (2001). Genotypic variation for tolerance to phosphorus deficiency in rice and the potential for its exploitation in rice improvement. Plant Breed.

[CR181] Wissuwa M, Ae N (2001). Further characterization of two QTLs that increase phosphorus uptake of rice (*Oryza sativa* L.) under phosphorus deficiency. Plant Soil.

[CR182] Wissuwa M, Yano M, Ae N (1998). Mapping of QTLs for phosphorusdeficiency tolerance in rice (*Oryza sativa* L.). Theor Appl Genet.

[CR183] World Food Programme (2015) Types of malnutrition. http://www.wfp.org/hunger/malnutrition/types

[CR184] Wu P, Ni JJ, Luo AC (1998). QTLs underlying rice tolerance to lowpotassium stress in rice seedlings. Crop Sci.

[CR185] Xu X, Bai G (2015). Whole-genome resequencing: changing the paradigms of SNP detection, molecular mapping and gene discovery. Mol Breed.

[CR05] Xu JH, Messing J (2009). Amplification of prolamin storage protein genes in different subfamilies of the *Poaceae*. Theor Appl Genet.

[CR186] Yafang S, Gan Z, Jinsong B (2011). Total phenolic content and antioxidant capacity of rice grains with extremely small size. Afr J Agric Res.

[CR187] Yano K, Yamamoto E, Aya K, Takeuchi H, Lo PC, Hu L, Yamasaki M, Yoshida S, Kitano H, Hirano K, Matsuoka M (2016). Genome-wide association study using whole-genome sequencing rapidly identifies new genes influencing agronomic traits in rice. Nat Genet.

[CR188] Yawadio R, Tanimori S, Morita N (2007). Identification of phenolic compounds isolated from pigmented rices and their aldose reductase inhibitory activities. Food Chem.

[CR189] Ye X, Al-Babili S, Kloti A, Zhang J, Lucca P, Beyer P, Potrykus I (2000). Engineering the provitamin A (beta-carotene) biosynthetic pathway into (carotenoid-free) rice endosperm. Science.

[CR190] Yongmei G, Ping M, Jiafu L, Yixuan L, Zichao L (2007). QTL mapping and Q X E interactions of grain cooking and nutrient qualities in rice under upland and lowland environments. Acta Genet Sin.

[CR191] Yoshimura A, Ideta O, Iwata N (1997). Linkage map of phenotypeand RFLP markers in rice. Plant Mol Biol.

[CR192] Yu YH, Li G, Fan YY, Zhang KQ, Min J, Zhu ZW, Zhuang JY (2009). Genetic relationship between grain yield and the contents of protein and fat in a recombinant inbred population of rice. J Cereal Sci.

[CR193] Yu H, Xie W, Li J, Zhou F, Zhang Q (2014). A whole-genome SNP array (RICE6K) for genomic breeding in rice. Plant Biotechnol J.

[CR194] Yun BW, Kim MG, Handoyo T, Kim KM (2014). Analysis of rice grain quality-associated quantitative trait loci by using genetic mapping. Am J Plant Sci.

[CR195] Zhang MW, Guo BJ, Peng ZM (2004). Genetic effects on Fe, Zn, Mn and P content in *indica* black pericarp rice and their genetic correlations with grain characteristics. Euphytica.

[CR196] Zhang MW, Guo BJ, Zhang RF, Chi JW, Wei ZC, Xu ZH, Zhang Y, Tang XJ (2006). Separation, purification and identification of antioxidant compositions in black rice. Agri Sci China.

[CR197] Zhang W, Bi J, Chen L, Zheng L, Ji S, Xia Y, Xie K, Zhao Z, Wang Y, Liu L, Jiang L, Wan J (2008). QTL mapping for crude protein and protein fraction contents in rice (*Oryza sativa* L.). J Cereal Sci.

[CR198] Zhang YD, Zhang YH, Dong SL, Chen T, Zhao QY, Zhu Z, Zhou LH, Yao S, Zhao L, Yu X, Wang C (2013). QTL mapping for grain size traits based on extra-large grain rice line TD70. Rice Sci.

[CR199] Zhang M, Pinson SRM, Tarpley L, Huang XY, Lahner B, Yakubova E, Baxter I, Guerinot ML, Salt DE (2014). Mapping and validation of quantitative trait loci associated with concentrations of 16 elements in unmilled rice grain. Theor Appl Genet.

[CR06] Zhao BR, Xing QH, Xia HA, Yang HH, Jin DM, Liu X, Wang SW, Wang B, Yuan LP (2005). DNA Polymorphism among Yewei B, V20B and Oryza minuta J. S. Presl. ex CB Presl. J Integr Plant Biol.

[CR200] Zhao K, Tung CW, Eizenga GC, Wright MH, Ali ML, Price AH, Norton GJ, Islam MR, Reynolds A, Mezey J, McClung AM, Bustamante CD, McCouch SR (2011). Genomewide association mapping reveals a rich genetic architecture of complex traits in *Oryza sativa*. Nat Comm.

[CR201] Zheng L, Cheng Z, Ai C, Jiang X, Bei X, Zheng Y, Glahn RP, Welch RM, Miller DD, Lei XG, Shou H (2010). Nicotianamine, a novel enhancer of rice iron bioavailability to humans. PLoS ONE.

[CR202] Zhong M, Wang L, Yuan J, Luo L, Xu C, He YQ (2011). Identification of QTL affecting protein and amino acid contents in rice. Rice Sci.

[CR203] Zhou Z, Robards K, Helliwell S, Blanchard C (2004). The distribution of phenolic acids in rice. Food Chem.

[CR204] Zimmerman M, Hurrel R (2002). Improving iron, zinc and vitamin A nutrition through plant biotechnology. Curr Opin Biotechnol.

